# Exotic garden plants partly substitute for native plants as resources for pollinators when native plants become seasonally scarce

**DOI:** 10.1007/s00442-020-04785-8

**Published:** 2020-10-20

**Authors:** Michael Staab, Maria Helena Pereira-Peixoto, Alexandra-Maria Klein

**Affiliations:** 1 Department of Nature Conservation and Landscape Ecology, University of Freiburg, Tennenbacher Straße 4, 79106 Freiburg, Germany; 2 Freiburg Institute of Advanced Studies (FRIAS), University of Freiburg, Albertstraße 19, 79104 Freiburg, Germany; 3 CAPES Foundation, Ministry of Education of Brazil, Brasília, DF 70040-020 Brazil

**Keywords:** Bees, Flower visitors, Non-native species, Seasonal shift, Networks

## Abstract

Urban green spaces such as gardens often consist of native and exotic plant species, which provide pollen and nectar for flower-visiting insects. Although some exotic plants are readily visited by pollinators, it is unknown if and at which time of the season exotic garden plants may supplement or substitute for flower resources provided by native plants. To investigate if seasonal changes in flower availability from native vs. exotic plants affect flower visits, diversity and particularly plant–pollinator interaction networks, we studied flower-visiting insects over a whole growing season in 20 urban residential gardens in Germany. Over the course of the season, visits to native plants decreased, the proportion of flower visits to exotics increased, and flower-visitor species richness decreased. Yet, the decline in flower-visitor richness over the season was slowed in gardens with a relatively higher proportion of flowering exotic plants. This compensation was more positively linked to the proportion of exotic plant species than to the proportion of exotic flower cover. Plant–pollinator interaction networks were moderately specialized. Interactions were more complex in high summer, but interaction diversity, linkage density, and specialisation were not influenced by the proportion of exotic species. Thus, later in the season when few native plants flowered, exotic garden plants partly substituted for native flower resources without apparent influence on plant–pollinator network structure. Late-flowering garden plants support pollinator diversity in cities. If appropriately managed, and risk of naturalisation is minimized, late-flowering exotic plants may provide floral resources to support native pollinators when native plants are scarce.

**Electronic supplementary material:**

The online version of this article (10.1007/s00442-020-04785-8) contains supplementary material, which is available to authorized users.

## Introduction

Flying insects, including many wild pollinator species, have been dramatically declining in Europe (Hallmann et al. 2017; Powney et al. 2019). While these declines have been documented in agricultural and forest areas (Seibold et al. 2019), urban areas may retain pollinator diversity (Baldock et al. 2015; Sirohi et al. 2015; Wenzel et al. 2020). Thus, strategies to preserve pollinating insects and related ecosystem functions in urban areas were recognized as important (Threlfall et al. 2015; Hall et al. 2017; Theodorou et al. 2020). Residential gardens, in particular, can contain high plant diversity and contribute to the maintenance of urban biodiversity and pollinators (Gaston et al. 2005; Smith et al.^2006^; Quistberg et al. 2016).

Compared to natural habitats, gardens usually contain a high proportion of exotic ornamental plant species (Loram et al. 2008). The general value of flowering garden plants for supporting urban pollinators is unquestionable. Several studies have distinguished among the geographic origin (native vs. exotic) of garden plants (e.g. Hanley et al. 2014; Salisbury et al. 2015; Majewska et al. 2018; Frankie et al. 2019). For example, gardens with more native plants are associated with a greater abundance of native wild bees (Pardee and Philpott 2014). Due to the shared evolutionary history of native plants and pollinators, and the expected trait-based reciprocal plant–pollinator interactions (Jordano et al. 2003; Schleuning et al. 2015), it is assumed that native plants provide suitable resources for native pollinators (Morandin and Kremen 2013). This view is, however, controversial (Kendle and Rose 2000; Schlaepfer et al. 2011), as some pollinators have high visitation rates to exotic species (e.g. Hanley et al. 2014; Rollings and Goulson 2019). Whether native pollinators utilize exotic plants can depend on specialisation. While generalist species (e.g. polylectic bees) that dominate in urban areas (Wenzel et al. 2020) are expected to readily utilize resources from exotic plants (but see Tallamy et al. 2010), specialists (e.g. monolectic bees) may be restricted to their native host species (Schweiger et al. 2010; compare also Burghardt et al. 2010).

Exotic plants threaten natural ecosystems if they become invasive, which might displace native plants and alter plant communities (Memmott et al. 2005; Litt et al. 2014; van Kleunen et al. 2015) with possible subsequent reductions in the abundance and species richness of insects and other animals (e.g. Burghardt et al. 2010; Tallamy et al. 2010; Narango et al. 2018; Vanbergen et al. 2018). However, in a changing world, pollinator abundance and species richness in urban gardens often depend on plant species richness and flower availability independently of the geographic origin (e.g. Smith et al. 2006; Scriven et al. 2013; Salisbury et al. 2015; Wenzel et al. 2020). For example, Salisbury et al. (2015) showed that generalist native bees can benefit from exotic plants. Thus, in situations when native garden plants are sparse, pollen and nectar from exotic plants may provide an important substitute for native floral resources. This may happen when the relative proportion of native vs. exotic plants in gardens changes during the season (Frankie et al. 2019).

Many plants in gardens flower only for a short time. To provide flowering plants throughout the season, exotic plants are frequently selected to complement native plants (Niemelä et al. 2013). Thus, the proportion of native and exotic plants among the total flowering plant community is likely not constant, which, in turn, may affect pollinators. In non-tropical climates, native flower availability is highest in the beginning of the growing season (Salisbury et al. 2015; Frankie et al. 2019), suggesting that late-flowering exotic plants in gardens could be important for pollinators to supplement otherwise scarce resources (sensu Ogilvie and Forrest 2017; Timberlake et al. 2019). However, from past research, it is unclear if these changes in relative seasonal availability of native vs. exotic plants affect plant–pollinator interaction networks.

Changes in interaction networks (reviewed in Dormann et al. 2009) allow direct conclusions on how habitat conditions affect trophic interactions (e.g. Ballantyne et al. 2015; Staab et al. 2015; Pereira-Peixoto et al. 2016). In pollination networks, specialized species usually interact with few species while generalists interact with many (Jordano et al. 2003). If exotic plant species change the availability and suitability of nectar and pollen resources over the course of the season, this might directly affect network properties. Consequently, interaction networks are suitable tools to assess community-wide effects of exotic plant species on pollinators (Memmott and Waser 2002; Bartomeus et al. 2008; Valdovinos et al. 2009; Lowenstein et al. 2019). Exotic plant species might change networks by creating new interactions or weakening the strength of existing interactions (Russo et al. 2014), but empirical data are mixed. For example, Memmott and Waser (2002) found that even though over one-third of native pollinators visited exotic plant species, these attracted fewer pollinator species than native plants, resulting in less connected networks. In contrast, exotic plants are also known to promote generalized plant-pollinator interactions on agricultural land (Marrero et al. 2017). Exotic plants are frequently well-integrated into the core of pollination networks (Bartomeus et al. 2008; Russo et al. 2019) and interactions can be resistant to the removal or addition of exotic plants, indicating that plant origin does not necessarily influence network specialization and connectivity (e.g. Valdovinos et al. 2009; Russo et al. 2019). To the best of our knowledge, it remains to be tested if seasonal changes in the availability of native vs. exotic flowering plant species in gardens affect interaction diversity, linkage density and specialization of plant-pollinator interaction networks. For urban gardens that are dominated by generalist flower visitors (synthesized in Wenzel et al. 2020) able to interact with many plant species, we expected that these network properties will be invariant to seasonal changes in the proportion of exotic plant species.

Using a replicated study design in urban gardens, we addressed the following questions: (1) Does the proportion of exotic plants change over the course of the growing season (from April to October)? (2) Are exotic plants supplementing resource availability for flower-visiting insects when native resources are seasonally lacking? (3) Are exotic garden plants affecting interaction diversity, linkage density and specialization of plant-pollinator interaction networks?

## Methods

### Study area and studied garden sites

The study was conducted in 20 private residential gardens in and near Freiburg, south-western Germany (47°60′′N, 7°51′′E). Freiburg is located between the Black Forest and the Upper Rhine Plain and has a population of approximately 230,000 inhabitants. The climate of the region is temperate and humid with a mean annual precipitation of 826 mm and a mean annual temperature of 11.8 °C (Wein et al. 2016).

To include a broad range of local contexts, the gardens were selected to cover a gradient of urbanisation, from highly urbanized in the city centre, to more rural at the city margin (Fig. S1). All gardens were located at least 1 km apart from each other. This radius was chosen to ensure independent samples since most wild bee species have shorter flight distances (Zurbuchen et al. 2010). Garden size was measured in satellite imagery (WorldView-2, 50 cm panchromatic resolution, recorded on 17 April 2013) using ArcGIS (ESRI 2011). Size of the connected garden area was determined as the size of the entire joint area of gardens in the direct neighbourhood, limited by roads, buildings or agricultural fields. To obtain a quantitative measure for urbanisation, all sealed areas (e.g. roads, buildings, paved surfaces) in a 1 km radius around the centre of each garden were marked in the same satellite imagery. For analysis, the relative sealed area was calculated (Table 1).

**Table 1:** Summary information of studied gardens

Property	Range_(min–max)_	Mean ± SD
Garden size [m^2^]	99–3286	831 ± 848
Green space size [m^2^]	99–15,270	4963 ± 3873
Sealed area [%]	12.6–48.4	29.5 ± 12.4
Flower cover [%]	0.4–7.4	2.6 ± 2.0
Proportion exotic flower cover [%]	1.7–65.0	25.7 ± 18.4
Number of plant species in flower	7–60	31 ± 14
Proportion exotic flowering plant species [%]	13.6–45.7	31.6 ± 9.8
Number of flower visits	60–579	266 ± 142
Proportion visits to exotic flowers [%]	3.1–63.7	22.3 ± 15.5
Number of flower-visitor species	14–55	35 ± 13

Range and mean (± SD) for each value are given

### Garden plants and pollinator observations

Gardens were ornamental (i.e. no vegetable gardens) and their management varied widely: some gardens were mown and weeded regularly while others were managed less intensively (see Table 1; Fig. S2). All gardens had a wide range of native and exotic plant species and contained one to two apple trees (*Malus domestica*, cultivars with a low height), which is typical for gardens in southern Germany where apple is a commonly planted fruit tree. Managed honey bees were not kept at any of the studied gardens. From April to October 2015, we conducted monthly flowering plant–pollinator observations, resulting in a total of 140 data points (20 gardens × 7 months). This sampling period was chosen to match the main activity period of flower-visiting insects in the study area. In each garden, the plants in flower were observed for flower-visiting insects as potential pollinators for 10 min per month. Interactions were always recorded simultaneously by two observers proficient with the local flora and entomofauna (MHPP, student helper). During observations, gardens were divided in equally observed subsections to cover all flowering plant species in a representative way. For apple trees, observations were constrained to the parts of the tree that could be visually observed and reached with an insect net. Other insect-pollinated trees were rare and sampled the same way as apple trees. Only interactions in which the flower-visiting insect was in contact with the flower were scored as flower visits. Common visitor species (e.g. honey bees, *Bombus* spp.) were directly identified in the field. Specimens of less conspicuous species were collected and determined with identification keys (Electronic Supplemental Material). All flower-visiting insects (except few Calliphoridae, Syrphidae and Lepidoptera) were determined to species (or morphospecies) (Table S1).

Observations took place between 9.00 am and 18.30 pm, and were restricted to dry, calm, and sunny weather (mean temperature during observations 22.5 ± 4.7 °C). In three cases (twice in May, once in June), no observations could be conducted, as no flowers were present in the respective garden (due to management actions shortly before scheduled data collection). Thus, our final dataset contains 137 individual plant-pollinator interaction matrixes.

After each flower-visitor observation, all plant species flowering in this garden were identified to species level (Electronic Supplemental Material, Table S1) and grouped into native or exotic plant species (using the German reference flora www.floraweb.de), even when no flower visitors were observed. To get a measure for flower availability and cover, the proportional area covered by flowers of each plant species (among the total garden area) was visually estimated (without using a quadrat) by the same person (MHPP). We follow Schroeder (1969) and Kowarik (2002) and use the year 1492 as a threshold for defining ‘native’ and ‘exotic’ plant species.

### Data analyses

All statistical analyses were performed with R 3.4.2 (R Core Team 2017), using the packages ‘lme4’ (Bates et al. 2015) and ‘glmmTMB’ (Brooks et al. 2017) for generalized linear mixed models (glmms), ‘piecewiseSEM’ (Lefcheck 2016) for path analysis and ‘bipartite’ (Dormann et al. 2009) for network analyses. Residuals of all glmms were inspected with the ‘DHARMa’ package (Hartig 2017). No deviations from the specified error distributions were detected. All regression models were based on data pooled per garden and month (*n* = 137 data sets, see above). Sampling dates were converted to ‘Julian day’, defined as the continuous count of days in the year to be used as a continuous variable for seasonality.

To test if the availability of flowers from native compared to exotic plant species changed over time, we used beta-regression glmms (package ‘glmmTMB’) with the proportional area (i.e. relative flower cover) covered by native and exotic flowers as response variables. Besides Julian day, flower cover might depend on the specific context of a garden. Thus, we added the proportion of sealed area in a 1 km buffer, garden size (log-transformed) and the size of the continuous green space a garden was part of (log-transformed) as fixed effects. All fixed effects were standardized (mean = 0, SD = 1) prior to analyses. As the gardens varied widely in respect to plant composition and management, we used garden identity as random effect to account for differences among gardens not covered by the fixed effects (models are listed in Table S2). To test if the proportion of exotic flower cover among total flower cover (response variable) changed with ongoing season, we also used a beta-regression glmm with the fixed effects Julian day, sealed area, garden size, and green space size. Garden identity was the random effect in this model.

A similar parallel analytical approach using Poisson models for count data and binomial models for proportions of exotics (package ‘lme4’) was identically applied to flower visits, flowering plant species and visited plant species. To gain an overview on general community patterns, the response variables total (native and exotic pooled) flower visits, flowering-plant species richness, visited flowering-plant species richness and flower-visitor species richness were analysed with Poisson glmms. Fixed effects were Julian day, sealed area, garden size, and green space size. Garden identity was treated as random effect. For models with the response variables flower visits and flower-visitor species richness, the proportion of exotic flower cover was additionally included as a fixed effect to test whether exotic plant species were more frequently visited than expected (based on their relative availability). Furthermore, for models with the response variables flowering-plant species richness (total, native, exotic) and visited flowering-plant species richness (total, native, exotic), total flower cover was added as fixed effect. This was done to account for the possible influence of the number of observations on plant species richness. For the same reason, we added total flower visits as a fixed effect to the model with flower-visitor species richness as a response variable (all models in Table S2).

As domesticated honey bees were the most common flower visitors in our data, we also analysed the response of the proportion of visits by species other than honey bees with a binomial glmm. For this model, fixed effects were Julian day, sealed area, garden size, green space size, and the proportion of exotic flower cover. Garden identity was treated as random effect. Because glmms with Poisson and binomial errors tend to be over-dispersed, an observation level random effect following Harrison (2014) was added to each model when it improved model fit (inspected with ‘DHARMa’).

The species richness of flower visitors in the temperate climate of the studied sites is expected to be highest in late spring and to decrease with the progression of the season (e.g. Bosch et al. 1997; Timberlake et al. 2019). At the same time, we hypothesized that exotic flowering plants may supplement resources for flower visitors, making it likely that flower-visitor species richness depends simultaneously on the proportion of exotic plants and flowers as well as total flower cover and total flowering-plant species richness. Furthermore, the glmm analyses suggested that garden size may influence flower-visitor species richness. To test for this conditionality and to disentangle the potentially interrelated relationships among variables explaining flower-visitor species richness over time, we built an a priori path model (Table S3). This model included direct paths from Julian day and garden size to flower-visitor species richness. Indirect paths from Julian day to flower-visitor species richness via the proportion of exotic flower cover, the proportion of exotic flowering plant species, total flower cover, and total flowering-plant species richness were included. From garden size, the model contained indirect paths on flower-visitor species richness via proportion of exotic flower cover and the proportion of exotic flowering plant species. Additional paths were from the proportion of exotic flower cover and the proportion of exotic flowering plant species on total flower cover and total flowering-plant species richness. The relation between the proportion of exotic flower cover and the proportion of exotic flowering plant species was modelled as correlated error. The same was done for the relation between total flower cover and total plant species richness. The path model was calculated with ‘piecewiseSEM’ using the same standardized data as the glmms and included garden identity as random effect. Model fit was assessed with a separation test following Shipley (2009).

To obtain measures of plant–flower visitor interactions, we calculated network indices with ‘bipartite’. Of the many postulated indices, we used Shannon interaction diversity, linkage density and specialization (H2′). Those three indices are based on quantitative interactions (Blüthgen et al. 2008) and are relatively insensitive to variations in network size. Interaction diversity is the network equivalent of Shannon diversity, describing diversity of links in a network (larger values indicating higher diversity). Linkage density is a measure for the weighted density of links per species averaged for plants and flower visitors (values are 1 or larger, with larger values indicating more interactions among species). H2′ is a measure of specialisation, with values standardized between 0 and 1, with larger values indicating higher specialisation.

To test for changes in network structure during the progress of the season and in dependency to the availability of exotic species, we used linear mixed models (lmms, package ‘lme4’; applying Satterthwaite-approximated degrees of freedom). Network indices (Shannon interaction diversity, linkage density, H2′) were the response variables (models in Table S4). The fixed effects (all standardized to mean = 0 and SD = 1) were proportion of exotic species, Julian day, sealed area, garden size, green space size, and the log-transformed number of visited flowers to account for variation in network size. As visual inspection of relationships between network indices and Julian day suggested non-linear relationships with a maximum in summer, we also calculated the same models with Julian day as second-order polynomial. These non-linear relationships had a superior fit for Shannon interaction diversity (ΔAIC = 40.2) and linkage density (ΔAIC = 14.0), and for those indices Julian day was treated as second-order polynomial. Residuals of all lmms where inspected for normality and variance homogeneity, which was met in all cases.

## Results

### Flower visitor and plant community

We observed a total of 5310 flower-visiting individuals comprising 163 species (Table S1). Among all flower visitors, bees were by far the most abundant (4588 individuals, 86.8%) and species-rich (117 species) taxon, with the European honey bee *Apis mellifera* as the most frequent bee species (54.9%). Native wild bees accounted for 31.9% of visits (there were no exotic bee species). Flies and wasps comprised 11% and 2.2% of the visits to the flowers, respectively. A total of 334 flowering plant species were found in the gardens, of which 187 were native and 147 exotic (for a list of plant and flower-visiting species see Table S1). Apple (*M. domestica*) was the native plant with the highest flower cover (48.8%) and dominated interactions in spring. In autumn, species of *Trifolium* (clover) and *Geranium* (cranesbill) contributed most to native flower cover. The ivy-leaved pelargonium *Pelargonium peltatum* (4.2%) was the exotic species with highest flower cover. Plant species with highest flower cover did not necessarily attract most flower visits. While *M. domestica* received highest flower visitation among the native plants (26.9%), the invasive giant golden rod *Solidago gigantea* was the most attractive exotic plant (4.1% of total visits) even though it contributed only 0.4% to flower cover (Table S1). Other highly visited exotics included *Anemone hupehensis* (1.5% visits, 0.8% cover) and *Rudbeckia fulgida* (1.2%, 1.5%).

### Seasonal changes of plants and their flower-visiting species

**Fig. 1:**
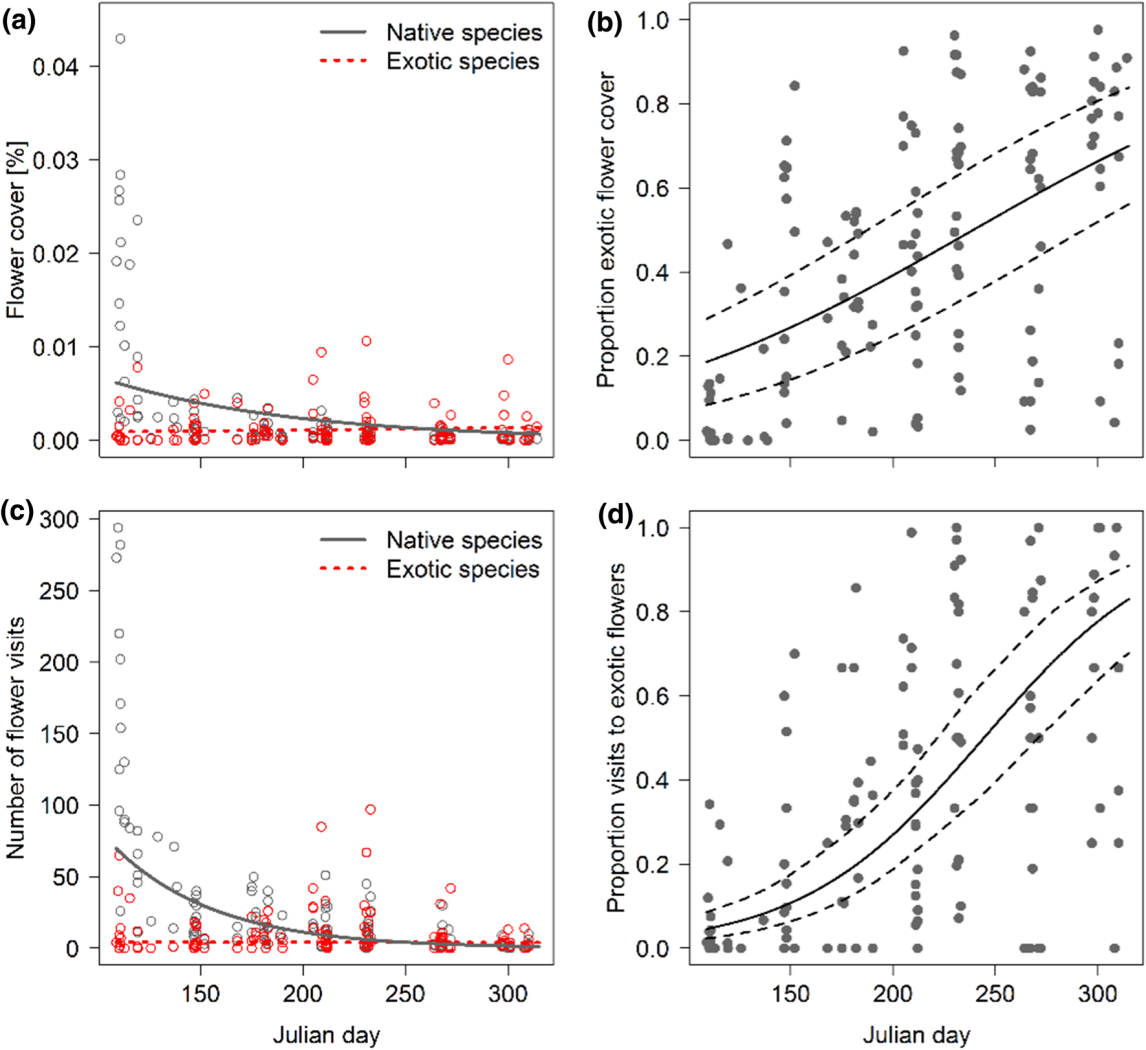
Flower availability (cover) and flower visits to native and exotic plant species change over the season. **a** The cover of native but not exotic flowers decreases from spring to fall. Simultaneously, **b** the proportion of exotic among total flower cover increases. **c** Absolute number of visits to native but not exotic flowers decreases likewise. As for flower availability, this results (**d**) in a higher proportion of visits to exotic flowers. Regression lines in (**a**) and (**b**) indicate the bootstrapped (*n* = 1000) predictions of beta glmms; in (**c**) and (**d**) the predictions (both *p* < 0.001, solid lines) of, respectively, a Poisson and a binomial glmm are shown. Solid lines are significant at *p* < 0.001, dotted lines are not significant, dashed lines indicate 95% CI

As expected, total flower cover (native and exotic pooled), total flower visits, total flowering-plant species richness and total visited plant species richness declined with the progressing season (see Table 2 and Table S2 for statistical details; Fig. S3). However, when native and exotic flowers were considered independently, only the cover of native flowers declined (*p* < 0.001, *z* = − 7.720), while the number of exotic flowers did not change (Fig. 1a). Simultaneously, the proportion of exotic among total flower cover increased (*p* < 0.001, *z* = 8.673; Fig. 1b). In April, around 5% of the flower cover consisted of exotic plant species but this proportion increased to, respectively, 54% and 66% in September and October. Patterns for the number of flower visits were similar: visits to native (*p* < 0.001, *z* = − 9.705) but not exotic plants decreased (Fig. 1c) and the proportion of visits to exotic flowers increased as the season progressed (*p* < 0.001, *z* = 3.919; Fig. 1d).

**Table 2:** Relationship between response variables and Julian day

Response	Type	Fixed effect	Estimate ± SE	*z*/*t*	*p* value
Flower cover					
Total flower cover	Beta	**Julian day**	**− 0.560 ± 0.084**	**− 6.641**	** < 0.001**
Exotic flower cover	Beta	Julian day	0.119 ± 0.065	1.834	0.067
Native flower cover	Beta	**Julian day**	**− 0.769 ± 0.010**	**− 7.720**	** < 0.001**
Proportion exotic flower cover	Beta	**Julian day**	**0.738 ± 0.085**	**8.673**	** < 0.001**
Flower visits					
Total flower visits	Poisson^a^	**Julian day**	**− 1.005 ± 0.102**	**− 9.821**	** < 0.001**
Visits to exotic flowers	Poisson^a^	Julian day	− 0.030 ± 0.118	− 0.251	0.802
Visits to native flowers	Poisson^a^	**Julian day**	**− 1.055 ± 0.109**	**− 9.705**	** < 0.001**
Proportion visits to exotic flowers	binomial^a^	**Julian day**	**0.653 ± 0.167**	**3.919**	** < 0.001**
Flowering-plant species richness					
Total flowering plant species	Poisson	**Julian day**	**− 0.062 ± 0.027**	**− 2.019**	**0.032**
Exotic flowering plant species	Poisson	**Julian day**	**0.284 ± 0.053**	**5.342**	** < 0.001**
Native flowering plant species	Poisson	**Julian day**	**− 0.196 ± 0.037**	**− 5.245**	** < 0.001**
Prop. exotic flow. plant species	Binomial	**Julian day**	**0.429 ± 0.055**	**7.775**	** < 0.001**
Visited plant species richness					
Total visited plant species	Poisson	**Julian day**	**− 0.231 ± 0.046**	**− 4.991**	** < 0.001**
Exotic visited plant species	Poisson	**Julian day**	**0.271 ± 0.077**	**3.510**	** < 0.001**
Native visited plant species	Poisson	**Julian day**	**− 0.471 ± 0.060**	**− 7.829**	** < 0.001**
Prop. visited exotic plant species	Binomial	**Julian day**	**0.720 ± 0.094**	**7.663**	** < 0.001**
Flower visitors					
Flower-visitor species richness	Poisson^a^	**Julian day**	**− 0.500 ± 0.083**	**− 6.041**	** < 0.001**
Proportion non-honey bee visits	Binomial^a^	**Julian day**	**0.664 ± 0.144**	**4.613**	** < 0.001**
Network indices					
Shannon interaction diversity	Linear	**Julian day**	**2.619 ± 0.383**	**6.838**_**(102.4)**_	** < 0.001**
		**Julian day^2**	**− 2.657 ± 0.367**	**− 7.250**_**(113.0)**_	** < 0.001**
Linkage density	Linear	**Julian day**	**1.936 ± 0.469**	**4.126**_**(113.0)**_	** < 0.001**
		**Julian day^2**	**− 1.841 ± 0.450**	**− 4.088**_**(113.0)**_	** < 0.001**
H2′	Linear	Julian day	0.025 ± 0.049	0.503_(92.0)_	0.616

Reported model parameters have been estimated with glmms (see ‘Type’) and are excerpts from the full models shown in Tables S2 and S4. *p* values of Poisson, binomial and beta models are based on *z* statistics (with each variable accounting for 1 df in the nominator of the 137 df in the denominator), *p* values for linear mixed models are based on *t* statistics of Satterthwaite-approximated degrees of freedom (given as subscript to *t* values). Significant relationships (at *p* < 0.05) are in bold

^a^Model with observation-level random effect

**Fig. 2:**
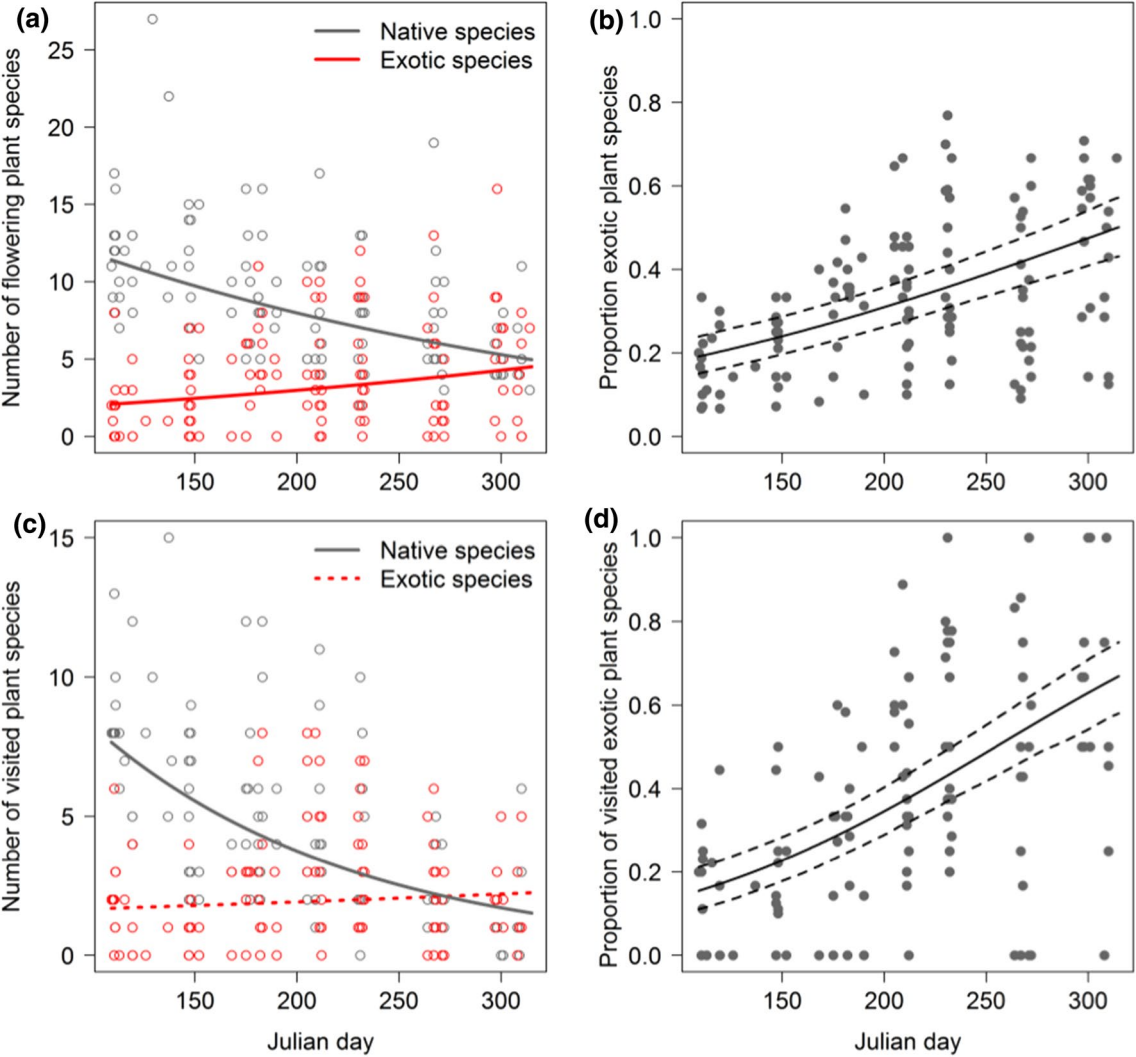
Absolute and relative numbers of flowering (i.e. plants in flower) and visited native and exotic plant species change over the season. **a** While the absolute number of native flowering plant species decreases from spring to fall, the number of exotic flowering plant species increases. **b** This results in an increasing proportion of exotic species that flower in the end of the season. In contrast to the plant species in flower, only the number of visited native but not exotic species is influenced by the season. Nevertheless, **d** the proportion of visited exotic plant species increases. Regression lines in (**a**) and (**c**) indicate the bootstrapped (*n* = 1000) predictions of Poisson glmms (solid lines are significant at *p* < 0.001; dotted lines are not significant); in (**b**) and (**d**) the predictions (both *p* < 0.001, solid lines) of binomial glmms with 95% CI (dashed lines) are shown

**Fig. 3:**
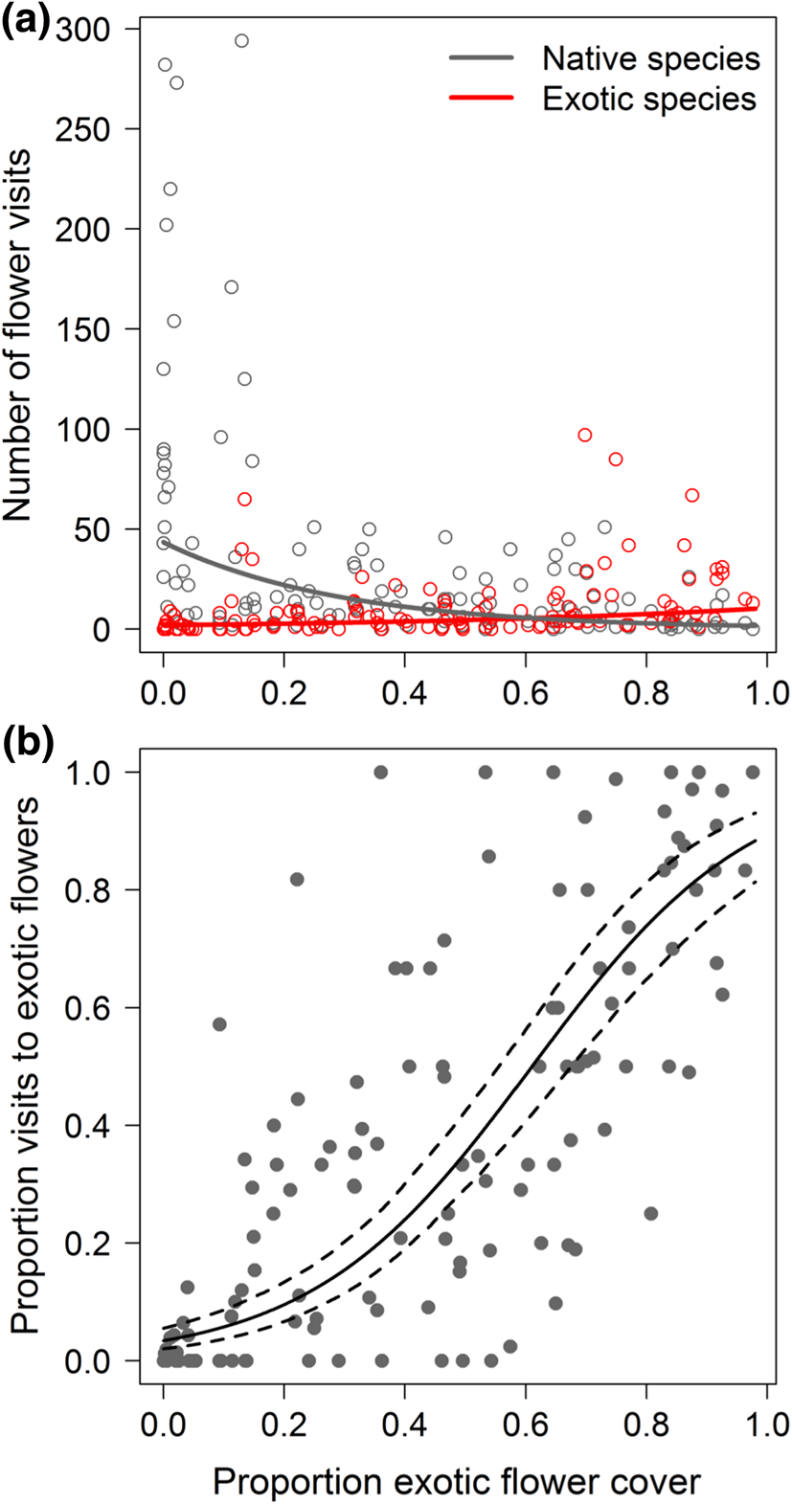
Absolute and relative number of flower visits to native and exotic plant species in response to the proportion of exotic among total flower cover. **a** While the absolute number of visits to native flowers decreases with increasing exotic flower cover, the number of visits to exotic flowers increases. **b** This results in a relative increase of visits to exotic flowers when exotic flower cover is high. Regression lines in (**a**) indicate bootstrapped (*n* = 1000) predictions of Poisson glmms (solid lines significant at *p* < 0.01); in (**b**) the prediction (*p* < 0.001) of a binomial glmm with 95% CI (dashed lines) is shown

The species richness of both native and exotic plants (in flower during data collection) was related to Julian day. While native flowering-plant species richness declined (*p* < 0.001, *z* = − 5.245), the number of flowering exotic plant species increased (*p* < 0.001, *z* = 5.342; Fig. 2a) as did the proportion of the flowering exotic plant species among all plant species in flower (*p* < 0.001, *z* = 7.775; Fig. 2b). The same patterns (all *p* < 0.001) occurred for the flowering plant species that were visited by insects (Fig. 2c, d). For example, less than 20% of all visited plant species were exotic in April but more than 50% in late summer and autumn. Visits to exotic flowers (*p* < 0.001, *z* = 5.220) increased and visits to native flowers decreased (*p* = 0.004, *z* = − 2.868; Fig. 3a) as the proportion of exotic flower cover increased (Table 3). Subsequently, the proportion of visits to exotic flowers increased with the proportion of exotic flower cover (*p* < 0.001, *z* = 6.840; Fig. 3b). Total flower visits were not related to exotic flower cover (Table 3). Sealed area and connected green space size had comparatively little explanatory power (Table S2). Larger gardens had generally lower flower cover, lower species richness of flowering exotic plants, and lower proportions of exotic flowering plant species and visited exotic plant species. Total and exotic plant species richness (for flowering and for visited plants) increased with total flower cover.

**Table 3:** Relationship between flower visits, flower visitors and proportion exotic flower cover

Response	Type	Fixed effect	Estimate ± SE	*z*	*p* value
Flower visits					
Total flower visits	Poisson^a^	Proportion exotic flower cover	0.113 ± 0.113	0.994	0.320
Visits to exotic flowers	Poisson^a^	**Proportion exotic flower cover**	**0.844 ± 0.162**	**5.220**	** < 0.001**
Visits to native flowers	Poisson^a^	**Proportion exotic flower cover**	− **0.327 ± 0.114**	− **2.868**	**0.004**
Proportion visits to exotic flowers	Binomial^a^	**Proportion exotic flower cover**	**1.220 ± 0.178**	**6.840**	** < 0.001**
Flower visitors					
Flower-visitor species richness	Poisson^a^	Proportion exotic flower cover	0.161 ± 0.094	1.763	0.084
Proportion non-honey bee visits	Binomial^a^	Proportion exotic flower cover	− 0.131 ± 0.136	− 0.966	0.334

Reported model parameters have been estimated with glmms (see ‘Type’) and are excerpts from the full models shown in Table S2. *p* values are based on *z* statistics. Significant relationships (at *p* < 0.05) are in bold. Each variable accounted for 1 df in the nominator of the 137 df in the denominator

^a^Model with observation-level random effect

Flower-visitor species richness was highest in spring and declined with season (*p* < 0.001, *z* = − 6.041; Fig. S4a). Flower-visitor species richness also increased with garden size (*p* = 0.033, *z* = 2.136) and total flower visits (*p* < 0.001, *z* = 3.792). Honey bees visited more flowers in spring and the proportion of visits by other species increased with Julian day (*p* < 0.001, *z* = 4.613; Fig. S4c). Neither flower-visitor species richness (Fig. S4b) nor visits by non-honey bees (Fig. S4d) were related to the proportion of exotic flower cover (Table 3).

**Fig. 4:**
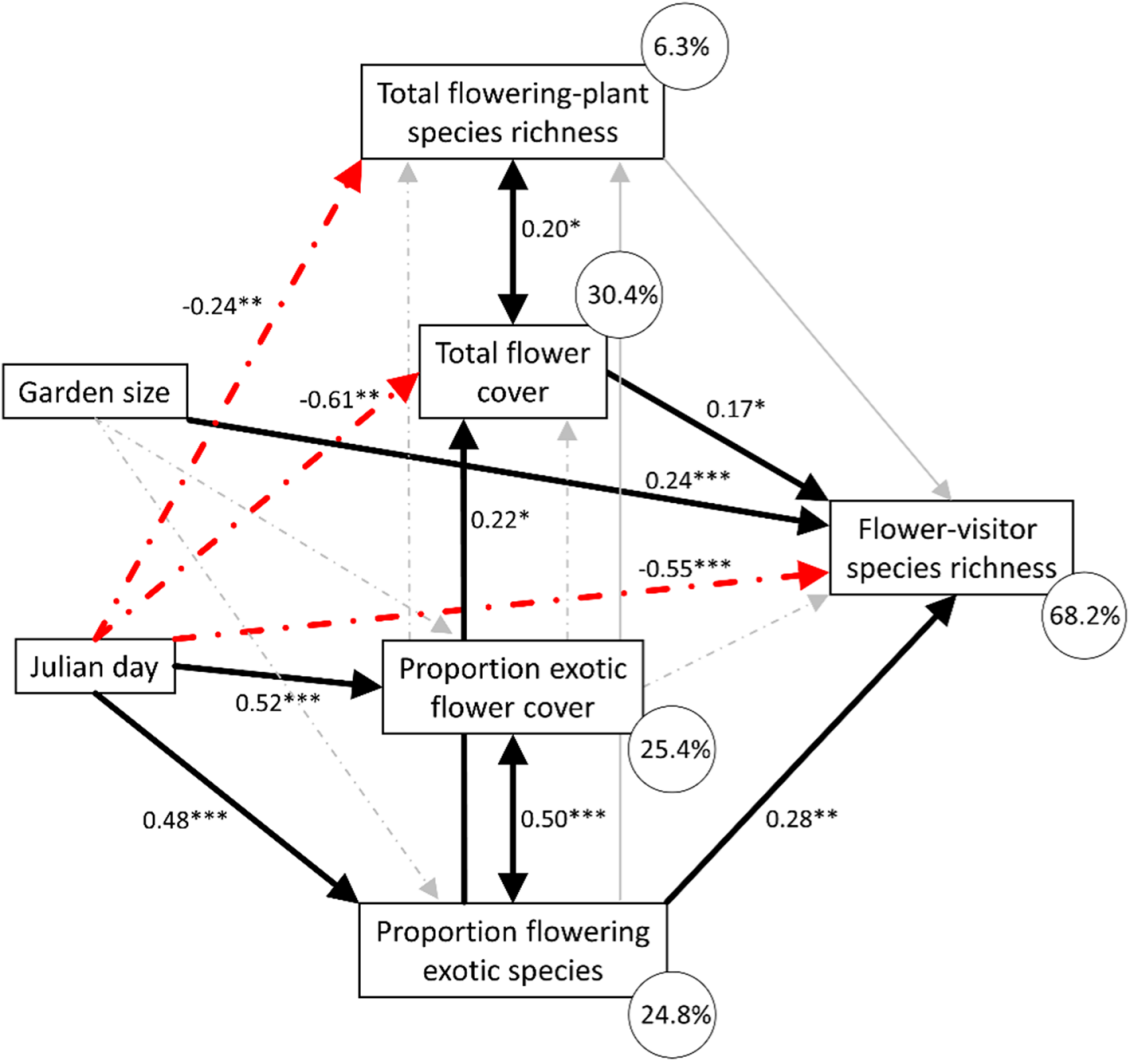
Path model (Fisher’s C = 4.94, *p* = 0.293) illustrating how direct and indirect relationships among the various interrelated variables influence flower-visitor species richness over the season. Numbers next to arrows are standardized path coefficients (*p < 0.05, **p < 0.01, ***p < 0.001). Solid arrows indicate positive relationship and dashed arrows indicate negative relationships. Correlated errors are indicated by double-headed arrows. Percentage values give explained marginal variances of endogenous variables. Thin arrows indicate non-significant relationships (with path coefficients omitted for clarity). Full numerical results are shown in Table S3

The general decline of flower-visiting species richness with season was confirmed by path analysis (Fig. 4). Additionally, the path model indicated that higher proportions of flowering exotic plant species partly compensate for the seasonal decline in flower-visitor species richness (*p* = 0.003; see Table S3 for full statistical details). In turn, the corresponding path from the proportion of exotic flower cover on flower-visitor species richness was not significant. This indicates that the seasonal increase in exotic flowering plant species affects flower visitors more via the proportion of exotic plant species than via the proportion of exotic flower cover. The proportion of exotic flowering plant species correlated positively with the proportion of exotic flower cover (correlated error, *p* < 0.001), and both variables were positively related to Julian day (*p* < 0.001). Furthermore, flower-visitor species richness in the path model increased with garden size (*p* < 0.001) and total flower cover (*p* < 0.021) but not total plant species richness (*p* = 0.309). Total flower cover and total plant species richness correlated positively (correlated error, *p* < 0.011) and decreased each over the season (*p* < 0.001). This a priori path model received high statistical support (Fisher’s C = 4.94, *p* = 0.293) and indicates that exotic flowering plants can indirectly mitigate the decline of pollinators during the season.

### Plant–flower-visitor interaction networks

**Fig. 5:**
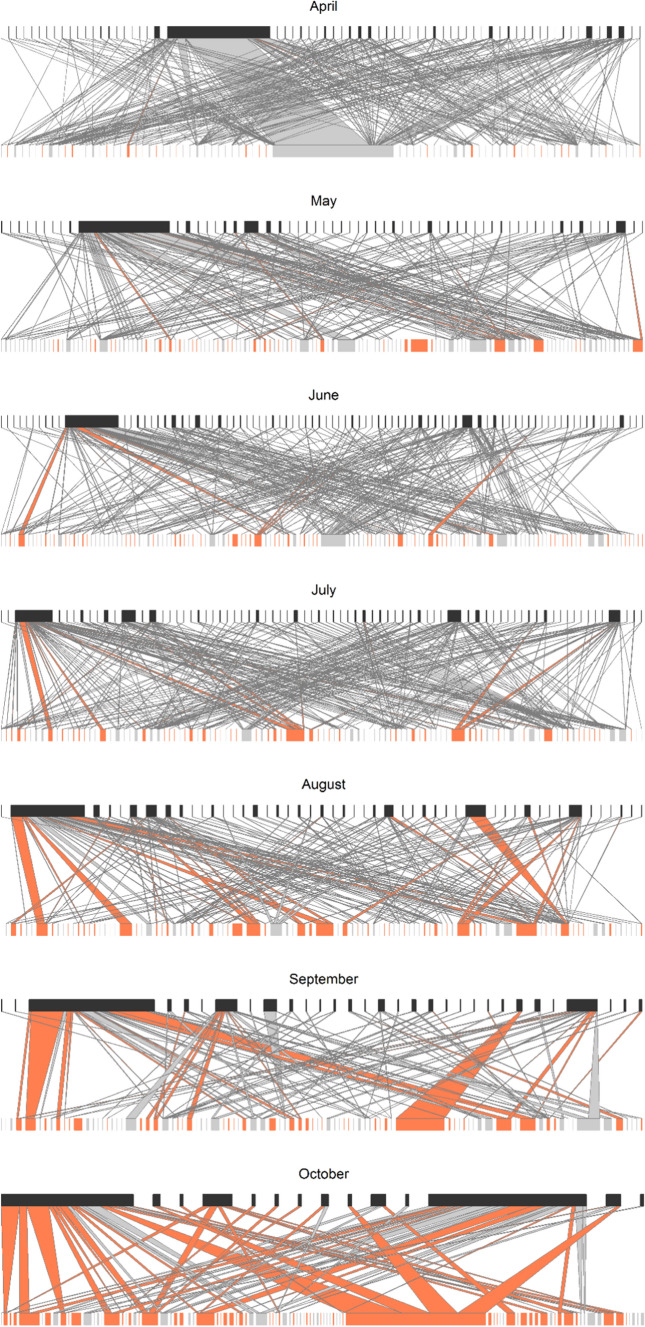
Seasonal changes in bipartite plant–pollinator networks, based on data pooled for all gardens per month. Width of bars corresponds to flower cover per plant species in the lower and to the number of visits by each flower-visiting species in the higher level; width of arrows corresponds to the number of interactions between two species, with the most narrow bars and arrows indicating single interactions each (note that the number of interactions varied: April = 2703, May = 525, June = 550, July = 657, August = 532, September = 237, October = 106). Arrows narrowing from top to bottom indicate that a plant species was more often visited than expected solely from the cover of this plant among all plants. In turn, arrows that widen from top to bottom indicate relatively less visited plant species. For plants, light grey bars and arrows indicate interactions of native plant species and red of exotic species, respectively. Non-visited plant species are included but do not have any interactions. While in April, most flowers were from native plants, this changed over the season. Networks with species codes are shown in Fig. S5

**Fig. 6:**
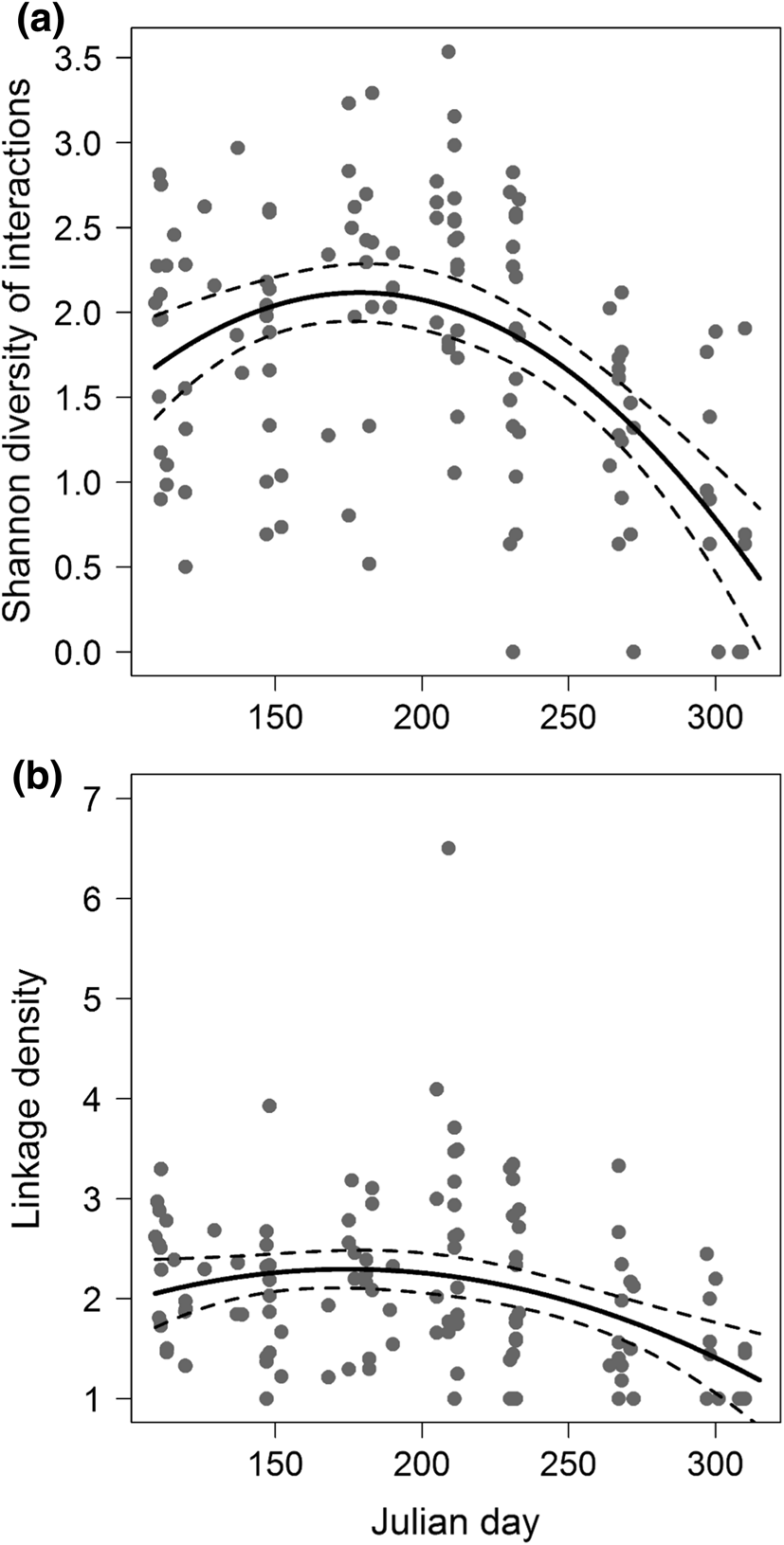
Seasonal changes in quantitative network indices. Both, **a** the Shannon diversity of species interactions and **b** the linkage density of networks peaked in summer. Regression lines indicate the bootstrapped (*n* = 1000) predictions of quadratic lmms (significant at *p* < 0.01) with 95% CI (dashed lines)

Plant–pollinator interactions per garden and month were moderately specialized (H2′ = 0.56 ± 0.28, mean ± SD). Overall, during each 10-min observation, plants and flower visitors interacted on average with two species each (LD = 2.05 ± 0.84) while identity of interacting plant species (native vs. exotic) shifted from spring to fall (Fig. 5; Fig. S5). Network indices except H2′ were positively correlated with the number of interactions in the network (expressed as number of total flower visits; see Table S4 for statistical details), which is expected, considering the mathematical properties of Shannon interaction diversity and linkage density. Interestingly, in addition to network size, both indices had an unimodal relationship with Julian day (*p* < 0.001; statistical details in Table 2), indicating more complex interactions in the mid of the season during high summer (Fig. 6a, b). H2′ was not related to Julian day and all network indices were independent of garden properties and the proportion of exotic species (Table S4).

## Discussion

### Exotic plants provide resources for flower visitors later in the season

We observed seasonal changes in flower visitation on native and exotic plant species in residential city gardens. Exotic plants gained importance for pollinators in high summer (June), which further increased until the end of the season. Similarly, flowering garden plants in the UK and Canada were shown to provide food resources for pollinators regardless of the plants’ origin (Salisbury et al. 2015; Martins et al. 2017). Our study goes beyond those earlier findings by highlighting that exotic plant species may functionally substitute for native garden plants and may maintain plant–pollinator interaction networks late in the season. Thus, exotic plants can provide resources for pollinators when flowering native plants become scarce. For example, generalist bees such as many *Bombus* spp. often do not discriminate between exotic and native plant species (Hanley et al. 2014). For those pollinators, exotic plants may ensure at least minimum pollen and nectar supply in otherwise resource poor periods (Timberlake et al. 2019). In our study, almost all flower visitors (including all bees) were native species. At the end of the season, visits to flowers of exotic plants dominated the interactions. Nevertheless, native bees may prefer the co-evolved native plant partners over the exotic plants, and visit exotics mostly when natives are scarce (Memmott and Waser 2002; Morandin and Kremen 2013; Frankie et al. 2019). Whether visitation to exotic plants influences plant (and likewise bee) fitness depends on the specific context (Vanbergen et al. 2018). For example, exotic plants that flower early in the season may reduce visitation to native plant species, which may increase the invasiveness of certain exotic species through reduced seed set of native plants (Morales and Traveset 2009). Testing for this potential competition (Levin and Anderson 1970; Bartomeus et al. 2008) is a promising topic for future research and would also help to answer whether exotic plants complement native flower visitors.

We found many locally endangered (Westrich et al. 2012) bee species visiting exotic plants. Compared to the high bee diversity, surprisingly few butterflies occurred in the studied gardens, which likely reflect the alarming population declines of butterflies in south-western Germany (Habel et al. 2019). In addition to flowers for adult feeding, butterflies also require suitable native host plants for larval development, and negative effects of exotic garden plants are common (Burghardt et al. 2009; Majewska et al. 2018). However, due to the paucity of butterfly records, we cannot test whether or not exotic plants have contributed to their scarcity. For bees, the planted exotic species, which are often from plant families represented in the native flora, can likely provide resources that are similar to the resources offered by native plants. This has been demonstrated specifically for other plant–pollinator (e.g. Buchholz and Kowarik 2019) and more generally for other plant–animal interactions (e.g. Wein et al. 2016). In urban areas generalist pollinators without inherent preferences for native or exotic plants are most common. These species forage on many plant species regardless of the plants’ geographic origin (Salisbury et al. 2015; Harrison and Winfree 2015; Wenzel et al. 2020). Thus, plant–pollinator networks in habitats dominated by exotic plants (such as gardens) can be structurally similar to natural areas (Valdovinos et al. 2009; Timóteo et al. 2018; Russo et al. 2019). The interaction networks observed by us were robust to the increase in exotic plants with progressing season, which may indicate substitution or supplementation of nectar and pollen by exotic plants when native plants are scarcely flowering. Thus, their time of presence may integrate exotic plants into local flower-visiting communities and interaction networks (Martins et al. 2017; Buchholz and Kowarik 2019).

### Seasonal changes of plant–pollinator interaction networks

Our study shows more diverse and complex interactions in high summer, which is likely related to a high heterogeneity of flower resources, since there is at this time an overlap between early and late-flowering species. Similarly, early and late-flying flower-visiting species overlap in this period, leading to more redundant interactions in the middle of the season. Independently of seasonality, exotic plants can shape interactions with bees in disturbed habitats (Williams et al. 2011), when a subset of highly visited exotic plant species may drive overall visitation patterns. In our data, pollinators visited exotic plants based on their availability, as the portion of visits to exotic plant species scaled with relative exotic flower cover and with the proportion of exotic species in flower. This was especially pronounced in the second half of the season, indicated by the relative increase in the exotic plants that were visited. Here, the path model combining the direct and indirect relationships among the plant variables suggested that effects of flowering exotic plant species were primarily mediated via the proportion of exotic species.

However, in accordance with Williams et al. (2011), exotic plants were not systematically preferred over natives. From the flower visitors’ point of view, the similarity in interaction diversity, linkage density, and specialization between early and late season suggests that exotic plant species substituted for their native counterparts (Moroń et al. 2019). This was especially the case in those gardens where late in the season no natives but some exotics were flowering. When native plants are not available, native flower visitors have to obtain their resources from the available exotic plants, regardless of preferences for native plants, which did in our case not lead to seasonal differences in networks. Exotic plants can be fully integrated within plant-pollinator networks (Bartomeus et al. 2008; Valdovinos et al. 2009), and thus contribute to the temporal stability of plant–pollinator interactions (Larson et al. 2016). This potentially neutral influence of exotic plant species may, however, quickly change when exotic pollinators are present in the network. Exotic bees usually prefer exotic plants (Frankie et al. 2019), and invasive pollinators can drastically alter interaction networks of native plants (Aizen et al. 2008; Valido et al. 2019). As exotic bees are often common in urban areas (e.g. Fitch et al. 2019), it is noteworthy that all bee species collected by us are native to Germany. It will be interesting to see if the likely spread of exotic bees in the area (Le Féon et al. 2018) will influence plant-pollinator interactions.

Notably, network structure was, in our study, not solely driven by resource availability, which is often the case in generalist networks (e.g. Dormann et al. 2009). The absolute number of flower visits in the studied gardens decreased, while the proportion of visited exotic plant species increased with the progression of the season. This also indicates that apple trees, which contributed substantially to total flower availability early in the season, were not biasing overall visitation patterns. Likewise, visitation patterns were not driven by honey bees, whose proportion among all visits declined with season but were unrelated to exotic flower cover. This may indicate that influences of exotic plants are similar among functional groups (honey bees vs. wild bees). Despite shifts in floral resource identity, networks maintained their interaction diversity, specialization, and linkage over time. Network properties were not different in spring compared to fall. Thus, exotic plants in our study seem not to compromise functional plant-pollinator network structure in gardens (Carman and Jenkins 2016), which also agrees with a recent synthesis of plant-pollinator networks (Timóteo et al. 2018). Based on these results, it may not be necessary to solely prioritize garden plants according to their origin when the aim is to bolster pollinators in urban areas, as some exotic plants can temporarily supplement native plant-pollinator interactions. However, plant–pollinator networks are known for their high spatial and temporal plasticity (Burkle and Alarcón 2011). As our study was restricted to a single area and one year, we do not know if our findings hold for other geographic settings and years. Furthermore, the lack of species-level identification for Syrphidae might influence our results, particularly late in the season when Syrphidae make up a notable proportion of flower visitors. Nevertheless, as in September and October interactions of Syrphidae were equally distributed among native and exotic plants, a large bias is unlikely.

### Ecological implications for garden management

While the deliberate planning of exotic plant species is highly controversial, exotics do not always have negative impacts. In habitat restoration projects (D'Antonio and Meyerson 2002), exotics can, for example, help to ameliorate soil fertility. In gardens, exotic plants are highly managed and could, late in the season, supplement native flowering plants to provide pollen and nectar for wild bees. Our results suggest that garden owners should plant natives that flower early in the season and may carefully consider to plant exotics that flower later when native plants are hardly available, so that constant flower resource availability is assured. This suggestion comes with a caveat, as exotic plants bear the risk of escaping from gardens and becoming invasive (van Kleunen et al. 2018). It is important to identify which exotic plants offer the least risk for invasion while extending the pollen and nectar supply for wild bees into the autumn (Tasker et al. 2020). In our data, gardened *S. gigantea*, despite being a globally invasive species, was attractive to many pollinators, although its total flower cover was low. In anthropogenically transformed but managed habitats such as urban gardens, similar but less invasive exotic plants may help to promote pollinators (Schlaepfer et al. 2011), while they may outcompete native vegetation and reduce pollinator diversity in more natural habitats such as meadows (e.g. Moroń et al. 2009). However, strategies to increase flower resources for urban pollinators should not primarily and exclusively target exotic plants, even though at present comparatively few late-flowering native species appeal to garden owners. For example, native *Geranium* and *Trifolium* species (also occurring partly naturally in gardens without being planted) flowered throughout September and October, and may together with other native species be used to increase resource availability for pollinators (Rundlöf et al. 2014).

Ideally such strategies for pollinators should be established at larger spatial scales, for example by including public green spaces in cities, because insects respond to resources at the landscape scale (e.g. Seibold et al. 2019). If large-scale actions are not feasible every individual garden owner can support pollinators by diversifying his or her garden. While many wild bees can forage at distances of up to 500 m or more (for large species) from their nest, this is energetically costly. Most individuals prefer to nest close-by flowers (Zurbuchen et al. 2010), which makes them benefit from local floral resources (e.g. Minckley et al. 1994). Combined with further insect-friendly measures such as avoidance of pesticides (exotic plant species often experience less pest problems), reduced mowing to allow some flowering weeds (Lowenstein et al. 2019), and the provision of nesting opportunities (von Königslöw et al. 2019), diverse pollinator communities may be preserved in highly modified urban ecosystems. To make sure that the late-flowering exotic plants have no negative effects on native pollinators, future studies might also consider effects of prolonged seasons due to climate change, where increased flower resources over consecutive years might influence fitness of individual pollinator species and consequently plant–pollinator interactions.

## Electronic supplementary material

Below is the link to the electronic supplementary material.

Supplementary file1 (DOCX 7590 kb)

## References

[CR1] Aizen MA, Morales CL, Morales JM (2008). Invasive mutualists erode native pollination webs. PLOS Biol.

[CR2] Baldock KC, Goddard MA, Hicks DM, Kunin W, Mitschunas N, Osgathorpe L, Memmott J (2015). Where is the UK’s pollinator biodiversity? The importance of urban areas for flower visiting insects. Proc R Soc Lond.

[CR3] Ballantyne G, Baldock KCR, Willmer PG (2015). Constructing more informative plant–pollinator networks: visitation and pollen deposition networks in a heathland plant community. Proc R Soc Lond.

[CR4] Bartomeus I, Vilà M, Santamaría L (2008). Contrasting effects of invasive plants in plant–pollinator networks. Oecologia.

[CR5] Bates D, Maechler M, Bolker BM, Walker SC (2015). Fitting linear mixed-effects models using lme4. J Stat Softw.

[CR6] Blüthgen N, Fründ J, Vazquez DP, Menzel F (2008). What do interaction network metrics tell us about specialization and biological traits?. Ecology.

[CR7] Bosch J, Retana J, Cerda X (1997). Flowering phenology, floral traits and pollinator composition in a herbaceous Mediterranean plant community. Oecologia.

[CR8] Brooks ME, Kristensen K, van Benthem KJ, Magnusson A, Berg CW, Nielsen A, Skaug HJ, Maechler M, Bolker BM (2017). glmmTMB balances speed and flexibility among packages for zero-inflated generalized linear mixed modeling. R J.

[CR9] Buchholz S, Kowarik I (2019). Urbanisation modulates plant pollinator interactions in invasive vs. native plant species. Sci Rep.

[CR10] Burghardt KT, Tallamy DW, Shriver WG (2009). Impact of native plants on bird and butterfly biodiversity in suburban landscapes. Conserv Biol.

[CR11] Burghardt KT, Tallamy DW, Philips C, Shropshire KJ (2010). Non-native plants reduce abundance, richness, and host specialization in lepidopteran communities. Ecosphere.

[CR12] Burkle LA, Alarcón R (2011). The future of plant-pollinator diversity: understanding interaction networks across time, space, and global change. Am J Bot.

[CR13] Carman K, Jenkins DG (2016). Comparing diversity to flower-bee interaction networks reveals unsuccessful foraging of native bees in disturbed habitats. Biol Conserv.

[CR14] D'Antonio C, Meyerson LA (2002). Exotic plant species as problems and solutions in ecological restoration: a synthesis. Restor Ecol.

[CR15] Dormann C, Fründ J, Blüthgen N, Gruber B (2009). Indices, graphs and null models: analyzing bipartite ecological networks. Open Ecol J.

[CR16] ESRI (2011). ArcGIS Desktop: Release 10.

[CR17] Fitch G, Wilson CJ, Glaum P, Chatura V, Simao MC, Jamieson MA (2019). Does urbanization favour exotic bee species? Implications for the conservation of native bees in cities. Biol Lett.

[CR18] Frankie G, Pawelek J, Chase MH, Jadallah CC, Feng I, Rizzardi M, Thorp R (2019). Native and non-native plants attract diverse bees to urban gardens in California. J Poll Ecol.

[CR19] Gaston K, Warren PH, Thompson K, Smith RM (2005). Urban domestic gardens (IV): the extent of the resource and its associated features. Biodivers Conserv.

[CR20] Habel JC, Trusch R, Schmitt T, Ochse M, Ulrich W (2019). Long-term large-scale decline in relative abundances of butterfly and burnet moth species across south-western Germany. Sci Rep.

[CR21] Hall DM (2017). The city as a refuge for insect pollinators. Conserv Biol.

[CR22] Hallmann CA (2017). More than 75 percent decline over 27 years in total flying insect biomass in protected areas. PLoS ONE.

[CR23] Hanley ME, Awbi AJ, Franco M (2014). Going native? Flower use by bumblebees in English urban gardens. Ann Bot.

[CR24] Harrison XA (2014). Using observation-level random effects to model overdispersion in count data in ecology and evolution. PeerJ.

[CR25] Harrison T, Winfree R (2015). Urban drivers of plant–pollinator interactions. Funct Ecol.

[CR26] Hartig F (2017) DHARMa: Residual diagnostics for hierarchical (multi-level /mixed) regression models. R package version 0.1.5. Available from https://CRAN.R-project.org/package=DHARMa

[CR27] Jordano P, Bascompte J, Olesen JM (2003). Invariant properties in coevolutionary networks of plant-animal interactions. Ecol Lett.

[CR28] Kendle AD, Rose JE (2000). The aliens have landed! What are the justifications for “native only” policies in landscape plantings?. Landsc Urban Plan.

[CR29] Kowarik I (2002). Biologische Invasionen in Deutschland: zur Rolle nichteinheimischer Pflanzen. Neobiota.

[CR30] Larson DL, Rabie PA, Droege S, Larson JL, Haar M (2016). Exotic plant infestation is associated with decreased modularity and increased numbers of connectors in mixed-grass prairie pollination networks. PLoS ONE.

[CR31] Le Féon V, Aubert M, Genoud D, Ponel-Andrieu V, Westrich P, Geslin B (2018). Range expansion of the Asian native giant resin bee *Megachile sculpturalis* (Hymenoptera, Apoidea, Megachilidae) in France. Ecol Evol.

[CR32] Lefcheck JS (2016). piecewiseSEM: Piecewise structural equation modelling in R for ecology, evolution, and systematics. Methods Ecol Evol.

[CR33] Levin DA, Anderson WW (1970). Competition for pollinators between simultaneously flowering species. Am Nat.

[CR34] Litt AR, Cord EE, Fulbright TE, Schuster GL (2014). Effects of invasive plants on arthropods. Conserv Biol.

[CR35] Loram A, Warren P, Gaston K (2008). Urban domestic gardens (XIV): the characteristics of gardens in five cities. Environ Manage.

[CR36] Lowenstein DM, Matteson KC, Minor ES (2019). Evaluating the dependence of urban pollinators on ornamental, non-native, and ‘weedy’ floral resources. Urban Ecosyst.

[CR37] Majewska AA, Sims S, Wenger SJ, Davis AK, Altizer S (2018). Do characteristics of pollinator-friendly gardens predict the diversity, abundance, and reproduction of butterflies?. Insect Conserv Divers.

[CR38] Marrero HJ, Torretta JP, Vázquez DP, Hodara K, Medan D (2017). Exotic plants promote pollination niche overlap in an agroecosystem. Agric Ecosyst Environ.

[CR39] Martins KT, Gonzalez A, Lechowicz MJ (2017). Patterns of pollinator turnover and increasing diversity associated with urban habitats. Urban Ecosyst.

[CR40] Memmott J, Waser NM (2002). Integration of alien plants into a native flower pollinator visitation web. Proc R Soc Lond.

[CR41] Memmott J, Alonso D, Berlow E, Dobson AP, Dunne JA, Sole RV, Weitz J, Dunne JA, Pascual M (2005). Habitat loss and food web structure. Ecological networks: linking structure to dynamics.

[CR42] Minckley RL, Wcislo WT, Yanega D, Buchmann SL (1994). Behavior and phenology of a specialist bee (*Dieunomia*) and sunflower (*Helianthus*) pollen availability. Ecology.

[CR43] Morales CL, Traveset A (2009). A meta-analysis of impacts of alien vs. native plants on pollinator visitation and reproductive success of co-flowering native plants. Ecol Lett.

[CR44] Morandin LA, Kremen C (2013). Hedgerow restoration promotes pollinator populations and exports native bees to adjacent fields. Ecol Appl.

[CR45] Moroń D, Lenda M, Skorba P, Szentgyörgyi H, Settele J, Woyciechowski M (2009). Wild pollinator communities are negatively affected by invasion of alien goldenrods in grassland landscapes. Biol Conserv.

[CR46] Moroń D, Skorka P, Lenda M (2019). Disappearing edge: the flowering period changes the distribution of insect pollinators in invasive goldenrod patches. Insect Conserv Divers.

[CR47] Narango DL, Tallamy DW, Marra PP (2018). Nonnative plants reduce population growth of an insectivorous bird. Proc Natl Acad Sci U S A.

[CR48] Niemelä J, Breuste JH, Elmqvist T, Guntenspergen G, James P, McIntyre NE (2013). Urban Ecology: Patterns Processes and Applications.

[CR49] Ogilvie JE, Forrest JRK (2017). Interactions between bee foraging and floral resource phenology shape bee populations and communities. Curr Opin Insect Sci.

[CR50] Pardee GL, Philpott SM (2014). Native plants are the bee’s knees: local and landscape predictors of bee richness and abundance in backyard gardens. Urban Ecosyst.

[CR51] Pereira-Peixoto MH, Pufal G, Staab M, Martins C, Klein AM (2016). Diversity and specificity of host-natural enemy interactions in an urban-rural interface. Ecol Entomol.

[CR52] Powney GD, Carvell C, Edwards M, Morris RKA, Roy HE, Woodcock BA, Isaac NJB (2019). Widespread losses of pollinating insects in Britain. Nat Commun.

[CR53] Quistberg RD, Bichier P, Philpott SM (2016). Landscape and local correlates of bee abundance and species richness in urban gardens. Environ Entomol.

[CR54] R Core Team (2017). R: A language and environment for statistical computing.

[CR55] Rollings R, Goulson D (2019). Quantifying the attractiveness of garden flowers for pollinators. J Insect Conserv.

[CR56] Rundlöf M, Persson AS, Smith HG, Bommarco R (2014). Late-season mass-flowering red clover increases bumble bee queen and male densities. Biol Conserv.

[CR57] Russo L, Memmott J, Montoya D, Shea K, Buckley YM (2014). Patterns of introduced species interactions affect multiple aspects of network structure in plant-pollinator communities. Ecology.

[CR58] Russo L, Albert R, Campbell C, Shea K (2019). Experimental species introduction shapes network interactions in a plant-pollinator community. Biol Invasions.

[CR59] Salisbury A, Armitage J, Bostock H, Perry J, Tatchell M, Thompson K (2015). Enhancing gardens as habitats for flower-visiting aerial insects (pollinators): should we plant native or exotic species?. J App Ecol.

[CR60] Schlaepfer MA, Sax DF, Olden JD (2011). The potential conservation value of non-native species. Conserv Biol.

[CR61] Schleuning M, Fründ J, Garcia D (2015). Predicting ecosystem functions from biodiversity and mutualistic networks: an extension of trait-based concepts to plant-animal interactions. Ecography.

[CR62] Schroeder FG (1969). Zur Klassifizierung der Anthropochoren. Vegetatio.

[CR63] Schweiger O (2010). Multiple stressors on biotic interactions: how climate change and alien species interact to affect pollination. Biol Rev.

[CR64] Scriven LA, Sweet MJ, Port GR (2013). Flower density is more important than habitat type for increasing flower visiting insect diversity. Inter J Ecol.

[CR65] Seibold S (2019). Arthropod decline in grasslands and forests is associated with landscape-level drivers. Nature.

[CR66] Shipley B (2009). Confirmatory path analysis in a generalized multilevel context. Ecology.

[CR67] Sirohi MH, Jackson J, Edwards M, Ollerton J (2015). Diversity and abundance of solitary and primitively eusocial bees in an urban centre: a case study from Northampton (England). J Insect Conserv.

[CR68] Smith RM, Gaston KJ, Warren PH, Thompson K (2006). Urban domestic gardens (VIII): environmental correlates of invertebrate abundance. Biodivers Conserv.

[CR69] Staab M, Blüthgen N, Klein AM (2015). Tree diversity alters the structure of a tri-trophic network in a biodiversity experiment. Oikos.

[CR70] Tallamy DW, Ballard M, D'Amico V (2010). Can alien plants support generalist insect herbivores?. Biol Invasions.

[CR71] Tasker P, Reid C, Young AD, Threfall CG, Latty T (2020). If you plant it, they will come: quantifying attractiveness of exotic plants for winter-active flower visitors in community gardens. Urban Ecosyst.

[CR72] Theodorou P (2020). Urban areas as hotspots for bees and pollination but not a panacea for all insects. Nat Commun.

[CR73] Threlfall CG, Walker K, Williams NSG, Hahs AK, Mata L, Stork N, Livesley SJ (2015). The conservation value of urban green space habitats for Australian native bee communities. Biol Conserv.

[CR74] Timberlake TP, Vaughan IP, Memmott J (2019). Phenology of farmland floral resources reveals seasonal gaps in nectar availability for bumblebees. J Appl Ecol.

[CR75] Timóteo S, O’Connor CJ, Lopez-Núnez FA, Costa JM, Gouveia AC, Heleno RH (2018). Pollination networks from natural and anthropogenic-novel communities show high structural similarity. Oecologia.

[CR76] Valdovinos FS, Ramos-Jiliberto R, Flores JD, Espinoza C, Lopez G (2009). Structure and dynamics of pollination networks: the role of alien plants. Oikos.

[CR77] Valido A, Rodriguez-Rodriguez MC, Jordano P (2019). Honeybees disrupt the structure and functionality of plant-pollinator networks. Sci Rep.

[CR78] van Kleunen M (2015). Global exchange and accumulation of non-native plants. Nature.

[CR79] van Kleunen M (2018). The changing role of ornamental horticulture in alien plant invasions. Biol Rev.

[CR80] Vanbergen AJ, Espíndola A, Aizen MA (2018). Risks to pollinators and pollination from invasive alien species. Nat Ecol Evol.

[CR81] von Königslöw V, Klein AM, Staab M, Pufal G (2019). Benchmarking nesting aids for cavity-nesting bees and wasps. Biodivers Conserv.

[CR82] Wein A, Bauhus J, Bilodeau-Gauthier S, Scherer-Lorenzen M, Nock C, Staab M (2016). Tree species richness promotes invertebrate herbivory on congeneric native and exotic tree saplings in a young diversity experiment. PLoS ONE.

[CR83] Wenzel A, Grass I, Belavadi VV, Tscharntke T (2020). How urbanization is driving pollinator diversity and pollination—a systematic review. Biol Conserv.

[CR84] Westrich P, Frommer U, Mandery K, Riemann H, Ruhnke H, Saure C, Voith J (2012). Rote Liste und Gesamtartenliste der Bienen (Hymenoptera, Apidae) Deutschlands. Naturschutz Biol Vielfalt.

[CR85] Williams N, Cariveau D, Winfree R, Cremen C (2011). Bees in disturbed habitats use, but do not prefer, alien plants. Basic Appl Ecol.

[CR86] Zurbuchen A, Lander L, Klaiber J, Müller A, Hein S, Dorn S (2010). Maximum foraging ranges in solitary bees: only few individuals have the capability to cover long foraging distances. Biol Conserv.

