# Supplementary material for: Exotic garden plants partly substitute for native plants as resources for pollinators when native plants become seasonally scarce
Source: Oecologia. 2020 Oct 20;194(3):465–80. doi: 10.1007/s00442-020-04785-8 (PMC7644476; doi:10.1007/s00442-020-04785-8)
Supplement: Supplementary file 1 — Supplementary file1 (DOCX 7590 kb) [file 442_2020_4785_MOESM1_ESM.docx]

**Electronic Supplemental Material**

Exotic garden plants partly substitute for native plants as resources for pollinators when native plants become seasonally scarce

Michael Staab, Maria Helena Pereira-Peixoto, Alexandra-Maria Klein

**Contents**

Table S1 List of plant and flower-visitor species…..................................................................................2

Table S2 Full model results for glmms........................................................................................................8

Table S3 Full results for the path model....................................................................................................11

Table S4 Full model results for network indices.....................................................................................12

Figure S1 Map of the study area..................................................................................................................13

Figure S2 Exemplary photographs of the studied gardens...................................................................14

Figure S3 Seasonal changes in the total plant communities................................................................15

Figure S4 Seasonal changes in the total flower-visitor communities...............................................16

Figure S5 Seasonal changes in total plant-pollinator networks..........................................................17

Literature used for species identification...................................................................................................19

**Table S1** List of the plant species in flower, their origin (n=native; e=exotic), flower-visitor species, their corresponding numerical codes for the interaction network graphics, and their abundance (i.e. the number of interactions per species)

| Plant species | Origin | Network number-plant | Abundance | Flower-visitor species | Network number-visitor | Abundance |
| --- | --- | --- | --- | --- | --- | --- |
| *Acer campestre* | n | 1 | 12 | *Ancistrocerus gazella* | 1 | 1 |
| *Acer japonicum* | e | 2 | 14 | *Ancistrocerus nigricornis* | 2 | 5 |
| *Achillea millefolium* | n | 3 | 50 | *Ancistrocerus parietum* | 3 | 1 |
| *Achillea ptarmica* | n | 4 | 1 | *Andrena* | 4 | 9 |
| *Aconitum carmichaelii* | e | 5 | 7 | *Andrena angustior* | 5 | 1 |
| *Adenophora confusa* | e | 6 | 9 | *Andrena carantonica* | 6 | 1 |
| *Aegopodium podagraria* | n | 7 | 10 | *Andrena chrysosceles* | 7 | 2 |
| *Agapanthus* sp. | e | 8 | 7 | *Andrena congruens* | 8 | 1 |
| *Ajuga reptans* | n | 9 | 102 | *Andrena dorsata* | 9 | 5 |
| *Alcea rosea* | e | 10 | 31 | *Andrena falsifica* | 10 | 3 |
| *Alcea* sp. | e | 11 | 5 | *Andrena flavipes* | 11 | 10 |
| *Alchemilla* sp. | n | 12 | 1 | *Andrena fulvago* | 12 | 1 |
| *Alchemilla vulgaris* | n | 13 | 14 | *Andrena haemorrhoa* | 13 | 8 |
| *Alliaria petiolata* | n | 14 | 12 | *Andrena harttofiana* | 14 | 1 |
| *Allium schoenoprasum* | n | 15 | 7 | *Andrena humilis* | 15 | 2 |
| *Allium* sp. | n | 16 | 15 | *Andrena lathyri* | 16 | 1 |
| *Allium ursinum* | n | 17 | 8 | *Andrena nitida* | 17 | 2 |
| *Althaea* sp. | n | 18 | 5 | *Andrena sericata* | 18 | 9 |
| *Alyssum saxatile* | n | 19 | 16 | *Andrena* sp. | 19 | 6 |
| *Alyssum* sp. | n | 20 | 1 | *Andrena spinigera* | 20 | 1 |
| *Amaranthus* sp. | e | 21 | 1 | *Andrena strohmella* | 21 | 5 |
| *Anagallis arvensis* | n | 22 | 4 | *Andrena subopaca* | 22 | 1 |
| *Anemone hupehensis* | e | 23 | 83 | *Andrena tarsata* | 23 | 1 |
| *Anemone* sp. | n | 24 | 1 | *Andrena vaga* | 24 | 2 |
| *Antirrhinum* sp. | e | 25 | 1 | *Andrena varians* | 25 | 1 |
| *Aquilegia buergeriana* | e | 26 | 1 | *Anthidium manicatum* | 26 | 6 |
| *Aquilegia sp.* | n | 27 | 1 | *Anthophora furcata* | 27 | 2 |
| *Aquilegia vulgaris* | n | 28 | 14 | *Anthophora plumipes* | 28 | 98 |
| *Arabis* sp. | n | 29 | 1 | Apidae sp.1 | 30 | 29 |
| *Arctium lappa* | n | 30 | 4 | Apidae sp.2 | 32 | 1 |
| *Arenaria* sp. | n | 31 | 1 | *Apis mellifera* | 29 | 2914 |
| *Aruncus dioicus* | n | 32 | 1 | Bethylidae sp. | 31 | 1 |
| *Aster novae-belgii* | e | 33 | 46 | Brachycera sp.1 | 33 | 3 |
| *Aster* sp. | n | 34 | 6 | Brachycera sp.2 | 67 | 31 |
| *Astilbe* sp. | e | 35 | 1 | Brachycera sp.3 | 68 | 1 |
| *Atriplex* sp. | n | 36 | 1 | Brachycera sp.4 | 162 | 1 |
| *Aubrieta deltoidea* | e | 37 | 1 | *Bombus* | 34 | 38 |
| *Aubrieta* Hybrid | e | 38 | 4 | *Bombus hortorum* | 35 | 16 |
| *Begonia* sp. | e | 39 | 10 | *Bombus humilis* | 36 | 7 |
| *Bellis perennis* | n | 40 | 340 | *Bombus hypnorum* | 37 | 16 |
| *Berberis vulgaris* | n | 41 | 108 | *Bombus lapidarius* | 38 | 52 |
| *Bergenia* sp. | e | 42 | 2 | *Bombus lucorum* | 39 | 15 |
| *Borago officinalis* | e | 43 | 26 | *Bombus pascuorum* | 40 | 175 |
| *Brunnera macrophylla* | e | 44 | 59 | *Bombus pratorum* | 41 | 60 |
| *Buddleja davidii* | e | 45 | 34 | *Bombus sylvarum* | 42 | 5 |
| *Buddleja* x *weyeriana* | e | 46 | 13 | *Bombus terrestris* | 43 | 111 |
| *Buxus sempervirens* | n | 47 | 3 | *Bombylius major* | 44 | 57 |
| *Calendula officinalis* | e | 48 | 8 | Calliphoridae | 46 | 48 |
| *Calendula* sp. | n | 49 | 4 | *Ceratina chalybea* | 47 | 1 |
| *Calluna* sp. | n | 50 | 6 | *Ceratina cucurbitina* | 48 | 1 |
| *Calluna vulgaris* | n | 51 | 3 | *Cerceris hortivaga* | 49 | 1 |
| *Caltha palustris* | n | 52 | 3 | *Cerceris rybyensis* | 50 | 4 |
| *Calystegia sepium* | n | 53 | 4 | *Chelostoma campanularum* | 51 | 12 |
| *Campanula carpatica* | e | 54 | 3 | *Chelostoma distinctum* | 52 | 1 |
| *Campanula patula* | n | 55 | 2 | *Chelostoma florisomne* | 53 | 4 |
| *Campanula poscharskyana* | e | 56 | 1 | *Chelostoma foveolatum* | 54 | 1 |
| *Campanula rotundifolia* | n | 57 | 39 | *Chelostoma rapunculi* | 55 | 6 |
| *Campanula* sp. | n | 58 | 2 | *Chelostoma* sp. | 56 | 1 |
| *Campsis* x *tagliabuana* | e | 59 | 11 | *Colletes hederae* | 57 | 20 |
| *Cardamine pratensis* | n | 60 | 90 | *Crossocerus annulipes* | 58 | 1 |
| *Catharanthus roseus* | e | 61 | 1 | *Crossocerus barbipes* | 59 | 1 |
| *Centaurea cyanus* | n | 62 | 13 | *Crossocerus palmipes* | 60 | 1 |
| *Centaurea jacea* | n | 63 | 2 | *Crossocerus* sp. | 61 | 1 |
| *Centaurea* sp. | n | 64 | 21 | Diprionidae sp. | 62 | 1 |
| *Cerastium holosteoides* | n | 65 | 19 | *Eristalis tenax* | 63 | 8 |
| *Chaenomeles* sp. | e | 66 | 2 | *Eucera longicornis* | 64 | 1 |
| *Chelidonium majus* | n | 67 | 10 | *Eucera nigrescens* | 65 | 9 |
| *Chrysanthemum* sp. | e | 68 | 2 | Eumeninae sp. | 66 | 1 |
| *Chrysanthemum* sp.1 | e | 69 | 12 | *Gasteruption* sp. | 69 | 4 |
| *Chrysanthemum* sp.2 | e | 70 | 2 | *Halictus confusus* | 70 | 5 |
| *Cichorium* intybus | n | 71 | 6 | *Halictus langobardicus* | 71 | 6 |
| *Cichorium* sp. | n | 72 | 1 | *Halictus maculatus* | 72 | 2 |
| *Cirsium arvense* | n | 73 | 18 | *Halictus scabiosae* | 73 | 3 |
| *Cirsium* sp. | n | 74 | 2 | *Halictus simplex* | 74 | 3 |
| *Clematis* sp. | n | 75 | 8 | *Halictus smaragdulus* | 75 | 2 |
| *Clematis* sp.2 | n | 76 | 1 | *Halictus subauratus* | 76 | 10 |
| *Clematis* sp.3 | n | 77 | 1 | *Halictus tumulorum* | 77 | 28 |
| *Clematis vitalba* | n | 78 | 3 | *Halicus subauratus* | 78 | 1 |
| *Clematis viticella* | e | 79 | 2 | *Heriades truncorum* | 79 | 11 |
| *Convolvulus arvensis* | n | 80 | 52 | *Hylaeus alpinus* | 80 | 2 |
| *Convolvulus tricolor* | e | 81 | 1 | *Hylaeus brevicornis* | 81 | 2 |
| *Conyza* sp. | e | 82 | 19 | *Hylaeus communis* | 82 | 38 |
| *Coreopsis* sp. | e | 83 | 1 | *Hylaeus confusus* | 83 | 2 |
| *Coriandrum sativum* | e | 84 | 7 | *Hylaeus gibbus* | 84 | 1 |
| *Corydalis lutea* | e | 85 | 3 | *Hylaeus gredleri* | 85 | 2 |
| *Cosmea* sp. | e | 86 | 3 | *Hylaeus hyalinatus* | 86 | 14 |
| *Cosmos bipinnatus* | e | 87 | 32 | *Hylaeus imperiallis* | 87 | 2 |
| *Crepis biennis* | n | 88 | 144 | *Hylaeus nigritus* | 88 | 1 |
| *Crepis capillaris* | n | 89 | 204 | *Hylaeus paulus* | 89 | 1 |
| *Cucumis sativus* | e | 90 | 1 | *Hylaeus punctatus* | 90 | 6 |
| *Cucurbita pepo* | e | 91 | 5 | *Hylaeus signatus* | 91 | 7 |
| *Cucurbita* sp. | e | 92 | 1 | *Hylaeus sp.* | 92 | 7 |
| *Cyclamen persicum* | e | 93 | 3 | *Hylaeus styriacus* | 93 | 3 |
| *Cydonia oblonga* | e | 94 | 7 | *Hylaeus taeniolatus* | 94 | 2 |
| *Cynoglossum amabile* | e | 95 | 1 | *Ichneumonidae* sp. | 95 | 12 |
| *Dahlia* sp. | e | 96 | 6 | *Isodontia mexicana* | 96 | 17 |
| *Daucus carota* | n | 97 | 20 | *Lasioglossum* | 97 | 83 |
| *Delphinium hibrido* | n | 98 | 4 | *Lasioglossum albipes* | 98 | 14 |
| *Deutzia* sp. | e | 99 | 8 | *Lasioglossum buccale* | 99 | 1 |
| *Dianthus barbatus* | e | 100 | 1 | *Lasioglossum calceatum* | 100 | 30 |
| *Dianthus carthusianorum* | n | 101 | 2 | *Lasioglossum costulatum* | 101 | 2 |
| *Dianthus caryophyllus* | e | 102 | 1 | *Lasioglossum cupromicans* | 102 | 2 |
| *Dianthus plumarius* | e | 103 | 1 | *Lasioglossum fulvicorne* | 103 | 10 |
| *Dianthus* sp. | n | 104 | 5 | *Lasioglossum glabriusculum* | 104 | 17 |
| *Digitalis purpurea* | n | 105 | 4 | *Lasioglossum griseolum* | 105 | 1 |
| *Echinops* sp. | n | 106 | 1 | *Lasioglossum laticepes* | 106 | 76 |
| *Echinops sphaerocephalus* | e | 107 | 12 | *Lasioglossum lativentre* | 107 | 2 |
| *Echium vulgare* | n | 108 | 15 | *Lasioglossum leucozonium* | 108 | 13 |
| *Enkianthus campanulatus* | e | 109 | 4 | *Lasioglossum lissonotum* | 109 | 1 |
| *Epimedium* sp. | e | 110 | 4 | *Lasioglossum lucidulum* | 110 | 1 |
| *Erica tetralix* | n | 111 | 2 | *Lasioglossum malachurum* | 111 | 6 |
| *Erigeron annuus* | e | 112 | 77 | *Lasioglossum marginatum* | 112 | 1 |
| *Erysimum cheiri* | n | 113 | 2 | *Lasioglossum morio* | 113 | 166 |
| *Eschscholtzia californica* | e | 114 | 2 | *Lasioglossum nigripes* | 114 | 1 |
| *Eupatorium cannabinum* | n | 115 | 12 | *Lasioglossum nitidulum* | 115 | 26 |
| *Eupatorium purpureum* | e | 116 | 2 | *Lasioglossum pauperatum* | 116 | 1 |
| *Euphorbia cyparissias* | n | 117 | 2 | *Lasioglossum pauxillum* | 117 | 48 |
| *Euphorbia hypericifolia* | e | 118 | 7 | *Lasioglossum politum* | 118 | 19 |
| *Euphorbia milii* | e | 119 | 1 | *Lasioglossum pygmaeum* | 119 | 3 |
| *Euphorbia* sp. | n | 120 | 1 | *Lasioglossum rufitarse* | 120 | 4 |
| *Felicia amelloides* | e | 121 | 1 | *Lasioglossum setulosum* | 121 | 1 |
| *Ficaria verna* | n | 122 | 7 | *Lasioglossum sexnotatum* | 122 | 1 |
| *Forsythia* x *intermedia* | e | 123 | 1 | *Lasioglossum* sp.1 | 123 | 1 |
| *Fragaria* sp. | n | 124 | 6 | *Lasioglossum* sp.2 | 124 | 1 |
| *Fragaria vesca* | n | 125 | 23 | *Lasioglossum* sp.3 | 125 | 1 |
| *Fuchsia regia* | e | 126 | 4 | *Lasioglossum tricinctum* | 126 | 1 |
| *Fuchsia* sp. | e | 127 | 11 | *Lasioglossum villosulum* | 127 | 5 |
| *Fuchsia* sp.2 | e | 128 | 1 | *Lasioglossum zonulum* | 128 | 5 |
| *Gaillardia aristata* | e | 129 | 1 | Lepidoptera sp.1 | 45 | 2 |
| *Galinsoga ciliata* | e | 130 | 7 | *Lestica clypeata* | 129 | 1 |
| *Galinsoga parviflora* | e | 131 | 1 | *Lucilia* sp. | 130 | 65 |
| *Galium album* | n | 132 | 1 | *Macroglossum stellatarum* | 131 | 16 |
| *Galium odoratum* | n | 133 | 2 | *Macropis europaea* | 132 | 1 |
| *Gaura lindheimeri* | e | 134 | 17 | *Macropis fulvipes* | 133 | 1 |
| *Gaura* sp. | e | 135 | 1 | *Megachile centuncularis* | 134 | 1 |
| *Geranium gracile* | n | 136 | 1 | *Megachile circumcinta* | 135 | 1 |
| *Geranium pratense* | n | 137 | 79 | *Megachile willughbiella* | 136 | 2 |
| *Geranium psilostemon* | e | 138 | 2 | *Melecta albifrons* | 137 | 2 |
| *Geranium pyrenaicum* | e | 139 | 9 | *Melitta haemorrhoidalis* | 138 | 1 |
| *Geranium robertianum* | n | 140 | 16 | *Mimumesa* sp. | 139 | 1 |
| *Geranium* sp. | n | 141 | 2 | *Musca domestica* | 140 | 64 |
| *Geranium* sp.1 | n | 142 | 82 | *Nitela* sp. | 141 | 1 |
| *Geranium sylvaticum* | n | 143 | 10 | *Nomada flavoguttata* | 142 | 1 |
| *Gerbera* sp. | e | 144 | 3 | *Nomada marshamella* | 143 | 1 |
| *Geum rivale* | n | 145 | 1 | *Nomada obtusifrons* | 144 | 1 |
| *Geum urbanum* | n | 146 | 1 | *Omalus* sp.1 | 145 | 1 |
| *Gladiolus* sp. | n | 147 | 1 | *Omalus* sp.2 | 146 | 1 |
| *Glandularia* sp. | e | 148 | 12 | *Osmia adunca* | 147 | 1 |
| *Glebionis coronaria* var. *discolor* | e | 149 | 1 | *Osmia bicolor* | 148 | 1 |
| *Glechoma hederacea* | n | 150 | 339 | *Osmia bicornis* | 149 | 111 |
| *Hedera helix* | n | 151 | 39 | *Osmia caerulescens* | 150 | 1 |
| *Helianthus annuus* | e | 152 | 19 | *Osmia cornuta* | 151 | 76 |
| *Helleborus niger* | n | 153 | 3 | *Osmia leaiana* | 152 | 1 |
| *Hemerocallis fulva* | e | 154 | 7 | *Oxybelus uniglumis* | 153 | 1 |
| *Heuchera sanguinea* | e | 155 | 4 | *Passaloecus* | 154 | 6 |
| *Hibiscus sp.* | e | 156 | 6 | *Passaloecus clypealis* | 155 | 1 |
| *Hibiscus syriacus* | e | 157 | 36 | *Passaloecus insignis* | 156 | 2 |
| *Hieracium aurantiacum* | n | 158 | 1 | *Passaloecus singularis* | 157 | 1 |
| *Hosta crispula* | e | 159 | 2 | *Pemphredon baltica* | 158 | 1 |
| *Hyacinthoides non-scripta* | n | 160 | 22 | *Pemphredon rugifer* | 159 | 1 |
| *Hydrangea macrophylla* | e | 161 | 52 | *Polistes dominula* | 160 | 6 |
| *Hydrangea* sp. | e | 162 | 1 | *Psenulus pallipes* | 161 | 1 |
| *Hypericum perforatum* | n | 163 | 25 | *Sapyga* sp. | 163 | 2 |
| *Iberis linifolia* | n | 164 | 1 | *Spilomena* sp. | 164 | 1 |
| *Iberis sempervirens* | e | 165 | 7 | *Stigmus pendulus* | 165 | 1 |
| *Iberis* sp. | n | 166 | 12 | Syrphidae | 166 | 298 |
| *Iberis umbellata* | e | 167 | 1 | *Tenthrodo* sp. | 167 | 1 |
| *Iris* x *germanica* | n | 168 | 2 | *Tetramorium caespitum* | 168 | 1 |
| *Iris* sp. | n | 169 | 3 | *Vespa crabro* | 169 | 4 |
| *Lagerstroemia* sp. | e | 170 | 1 | Vespidae sp. | 172 | 8 |
| *Lamium galeobdolon* | n | 171 | 25 | *Vespula germanica* | 170 | 27 |
| *Lamium* sp. | n | 172 | 18 | *Volucella zonaria* | 171 | 3 |
| *Lamprocapnos spectabilis* | e | 173 | 8 | *Xylocopa violacea* | 173 | 8 |
| *Lantana camara* | e | 174 | 2 |  |  |  |
| *Lathyrus pratensis* | n | 175 | 16 |  |  |  |
| *Lavandula angustifolia* | e | 176 | 78 |  |  |  |
| *Lavandula* sp. | e | 177 | 1 |  |  |  |
| *Leucanthemum* sp. | n | 178 | 1 |  |  |  |
| *Leucanthemum vulgare* | n | 179 | 61 |  |  |  |
| *Levisticum officinale* | e | 180 | 8 |  |  |  |
| *Lewisia cotyledon* | e | 181 | 5 |  |  |  |
| *Ligustrum vulgare* | n | 182 | 9 |  |  |  |
| *Lilium* sp.1 | n | 183 | 2 |  |  |  |
| *Lilium* sp.2 | n | 184 | 1 |  |  |  |
| *Linaria vulgaris* | n | 185 | 2 |  |  |  |
| *Linum grandiflorum* | e | 186 | 2 |  |  |  |
| *Linum* sp. | n | 187 | 2 |  |  |  |
| *Lobelia erinus* | e | 188 | 7 |  |  |  |
| *Lobelia richardii* | e | 189 | 3 |  |  |  |
| *Lobelia* x *gerardii* | e | 190 | 2 |  |  |  |
| *Lobularia maritima* | e | 191 | 3 |  |  |  |
| *Lonicera heckrottii* | e | 192 | 1 |  |  |  |
| *Lunaria annua* | e | 193 | 11 |  |  |  |
| *Lunaria* sp. | n | 194 | 1 |  |  |  |
| *Lysimachia vulgaris* | n | 195 | 35 |  |  |  |
| *Lythrum salicaria* | n | 196 | 5 |  |  |  |
| *Mahonia* sp. | e | 197 | 19 |  |  |  |
| *Malus domestica* | n | 198 | 1446 |  |  |  |
| *Malva moschata* | n | 199 | 11 |  |  |  |
| *Malva* sp. | n | 200 | 7 |  |  |  |
| *Matricaria chamomilla* | n | 201 | 10 |  |  |  |
| *Medicago lupulina* | n | 202 | 11 |  |  |  |
| *Medicago sativa* | e | 203 | 3 |  |  |  |
| *Melissa officinalis* | e | 204 | 52 |  |  |  |
| *Mentha* x *piperita* | n | 205 | 99 |  |  |  |
| *Mentha suaveolens* | n | 206 | 3 |  |  |  |
| *Muscari* sp. | n | 207 | 88 |  |  |  |
| *Myosotis arvensis* | n | 208 | 119 |  |  |  |
| *Myosotis* sp. | n | 209 | 7 |  |  |  |
| *Narcissus pseudonarcissus* | n | 210 | 9 |  |  |  |
| *Nepeta cataria* | n | 211 | 47 |  |  |  |
| *Nepeta* sp. | n | 212 | 1 |  |  |  |
| *Nerium oleander* | e | 213 | 15 |  |  |  |
| *Nigella damascena* | e | 214 | 1 |  |  |  |
| *Nigella sativa* | e | 215 | 3 |  |  |  |
| *Ocimum basilicum* | e | 216 | 5 |  |  |  |
| *Oenothera biennis* | e | 217 | 30 |  |  |  |
| *Origanum majorana* | e | 218 | 4 |  |  |  |
| *Origanum vulgare* | n | 219 | 47 |  |  |  |
| *Oxalis corniculata* | n | 220 | 1 |  |  |  |
| *Oxalis dillenii* | e | 221 | 5 |  |  |  |
| *Oxalis* sp.1 | n | 222 | 1 |  |  |  |
| *Oxalis* sp.2 | n | 223 | 4 |  |  |  |
| *Oxalis stricta* | e | 224 | 3 |  |  |  |
| *Paeonia* sp. | n | 225 | 5 |  |  |  |
| *Papaver rhoeas* | n | 226 | 10 |  |  |  |
| *Papaver* sp. | n | 227 | 1 |  |  |  |
| *Pelargonium peltatum* | e | 228 | 23 |  |  |  |
| *Pelargonium* sp. | e | 229 | 1 |  |  |  |
| *Petunia* sp. | e | 230 | 14 |  |  |  |
| *Phacelia tanacetifolia* | e | 231 | 7 |  |  |  |
| *Phalaenopsis* sp. | e | 232 | 1 |  |  |  |
| *Philadelphus* sp*.* | e | 233 | 3 |  |  |  |
| *Phlox* sp. | e | 234 | 18 |  |  |  |
| *Phlox subulata* | e | 235 | 10 |  |  |  |
| *Plantago lanceolata* | n | 236 | 4 |  |  |  |
| *Plantago major* | n | 237 | 29 |  |  |  |
| *Potentilla aurea* | n | 238 | 5 |  |  |  |
| *Potentilla fruticosa* | e | 239 | 16 |  |  |  |
| *Potentilla intermedia* | e | 240 | 1 |  |  |  |
| *Potentilla recta* | n | 241 | 12 |  |  |  |
| *Potentilla reptans* | n | 242 | 3 |  |  |  |
| *Potentilla* sp. | n | 243 | 1 |  |  |  |
| *Primula* sp. | n | 244 | 28 |  |  |  |
| *Primula veris* | n | 245 | 2 |  |  |  |
| *Prunella vulgaris* | n | 246 | 175 |  |  |  |
| *Prunus avium* | n | 247 | 42 |  |  |  |
| *Prunus domestica* | n | 248 | 15 |  |  |  |
| *Prunus laurocerasus* | e | 249 | 34 |  |  |  |
| *Pulmonaria officinalis* | n | 250 | 5 |  |  |  |
| *Ranunculus auricomus* | n | 251 | 3 |  |  |  |
| *Ranunculus lingua* | n | 252 | 16 |  |  |  |
| *Ranunculus repens* | n | 253 | 41 |  |  |  |
| *Ranunculus* sp. | n | 254 | 67 |  |  |  |
| *Ranunculus* sp.1 | n | 255 | 13 |  |  |  |
| *Ranunculus* sp.2 | n | 256 | 1 |  |  |  |
| *Raphanus raphanistrum* | n | 257 | 1 |  |  |  |
| *Rhododendron* sp. | n | 258 | 27 |  |  |  |
| *Ribes rubrum* | n | 259 | 28 |  |  |  |
| *Ribes sanguineum* | e | 260 | 50 |  |  |  |
| *Rosa rugosa* | e | 261 | 27 |  |  |  |
| *Rosa* sp. | n | 262 | 102 |  |  |  |
| *Rosmarinus officinalis* | e | 263 | 14 |  |  |  |
| *Rubus idaeus* | n | 264 | 54 |  |  |  |
| *Rubus* sect. *Rubus* | n | 265 | 155 |  |  |  |
| *Rudbeckia fulgida* | e | 266 | 76 |  |  |  |
| *Rudbeckia* sp. | e | 267 | 1 |  |  |  |
| *Rumex* sp. | n | 268 | 10 |  |  |  |
| *Sagina subulata* | n | 269 | 1 |  |  |  |
| *Salvia officinalis* | e | 270 | 17 |  |  |  |
| *Sambucus nigra* | n | 271 | 2 |  |  |  |
| *Sambucus* sp. | n | 272 | 1 |  |  |  |
| *Sanguisorba minor* | n | 273 | 2 |  |  |  |
| *Sanvitalia procumbens* | e | 274 | 2 |  |  |  |
| *Scorzoneroides autumnalis* | n | 275 | 17 |  |  |  |
| *Sedum acre* | n | 276 | 14 |  |  |  |
| *Sedum* sp. | n | 277 | 4 |  |  |  |
| *Sedum spectabile* | e | 278 | 41 |  |  |  |
| *Senecio erucifolius* | n | 279 | 12 |  |  |  |
| *Sinapis alba* | e | 280 | 10 |  |  |  |
| *Sinapis arvensis* | n | 281 | 5 |  |  |  |
| *Sinapis* sp. | n | 282 | 1 |  |  |  |
| *Solanum laxum* | e | 283 | 1 |  |  |  |
| *Solanum lycopersicum* | e | 284 | 2 |  |  |  |
| *Solanum melongena* | e | 285 | 1 |  |  |  |
| *Solanum nigrum* | n | 286 | 8 |  |  |  |
| *Solidago gigantea* | e | 287 | 222 |  |  |  |
| *Sonchus arvensis* | n | 288 | 7 |  |  |  |
| *Sonchus* sp. | e | 289 | 5 |  |  |  |
| *Spiraea* x *arguta* | e | 290 | 3 |  |  |  |
| *Stellaria media* | n | 291 | 16 |  |  |  |
| *Stellaria* sp. | n | 292 | 3 |  |  |  |
| *Symphytum officinale* | n | 293 | 35 |  |  |  |
| *Syringa* sp. | e | 294 | 10 |  |  |  |
| *Syringa vulgaris* | e | 295 | 18 |  |  |  |
| *Tagetes* sp. | e | 296 | 18 |  |  |  |
| *Tanacetum parthenium* | e | 297 | 9 |  |  |  |
| *Tanacetum* sp. | n | 298 | 2 |  |  |  |
| *Taraxacum officinale* | n | 299 | 401 |  |  |  |
| *Thymus* sp. | n | 300 | 1 |  |  |  |
| *Thymus vulgaris* | e | 301 | 7 |  |  |  |
| *Tibouchina* sp. | e | 302 | 1 |  |  |  |
| *Tibouchina urvilleana* | e | 303 | 1 |  |  |  |
| *Torilis arvensis* | n | 304 | 14 |  |  |  |
| *Trifolium arvense* | n | 305 | 1 |  |  |  |
| *Trifolium aureum* | n | 306 | 6 |  |  |  |
| *Trifolium pratense* | n | 307 | 102 |  |  |  |
| *Trifolium repens* | n | 308 | 110 |  |  |  |
| *Tropaeolum majus* | e | 309 | 18 |  |  |  |
| *Tropaeolum* sp. | e | 310 | 4 |  |  |  |
| *Tulipa* sp. | n | 311 | 50 |  |  |  |
| *Urtica* sp. | n | 312 | 4 |  |  |  |
| *Vaccinium myrtillus* | n | 313 | 4 |  |  |  |
| *Verbascum densiflorum* | n | 314 | 2 |  |  |  |
| *Verbascum thapsus* | n | 315 | 1 |  |  |  |
| *Verbena* sp. | n | 316 | 2 |  |  |  |
| *Veronica austriaca* | n | 317 | 1 |  |  |  |
| *Veronica chamaedrys* | n | 318 | 54 |  |  |  |
| *Veronica longifolia* | n | 319 | 11 |  |  |  |
| *Veronica persica* | e | 320 | 52 |  |  |  |
| *Veronica* sp. | n | 321 | 1 |  |  |  |
| *Viburnum opulus* | n | 322 | 3 |  |  |  |
| *Vicia cassubica* | n | 323 | 1 |  |  |  |
| *Vicia faba* | e | 324 | 1 |  |  |  |
| *Vicia lutea* | e | 325 | 2 |  |  |  |
| *Vicia sepium* | n | 326 | 55 |  |  |  |
| *Vinca minor* | n | 327 | 1 |  |  |  |
| *Vinca* sp. | n | 328 | 1 |  |  |  |
| *Viola odorata* | n | 329 | 1 |  |  |  |
| *Viola* sp. | n | 330 | 45 |  |  |  |
| *Virburnum* sp. | n | 331 | 15 |  |  |  |
| *Weigelia florida* | e | 332 | 10 |  |  |  |
| *Wisteria* sp. | e | 333 | 14 |  |  |  |
| *Yucca recurvifolia* | e | 334 | 1 |  |  |  |
|  |  |  |  |  |  |  |

**Table S2** Full model results of glmms testing for relationships between response variables and fixed effects (all standardized to mean = 0 and SD = 1; garden size and continuous green space size were log-transformed). Significant p-values are in bold. See also Table 2 and Table 3. Each variable accounted for 1 df in the nominator of the 137 df in the denominator

| Response | Type | Fixed effect | Estimate ± SE | z | p-value |
| --- | --- | --- | --- | --- | --- |
| *Flower availability* |  |  |  |  |  |
| Total flower cover | beta | **Julian day** | **-0.560 ± 0.084** | **-6.641** | **<0.001** |
|  |  | % sealed area | -0.136 ± 0.101 | -1.364 | 0.175 |
|  |  | **Garden size** | **-0.555 ± 0.109** | **-5.111** | **<0.001** |
|  |  | Green space size | 0.114 ± 0.090 | 1.270 | 0.204 |
|  |  |  |  |  |  |
| Exotic flower cover | beta | Julian day | 0.119 ± 0.065 | 1.834 | 0.067 |
|  |  | % sealed area | -0.252 ± 0.178 | -1.410 | 0.157 |
|  |  | **Garden size** | **-0.693 ± 0.190** | **-3.651** | **<0.001** |
|  |  | Green space size | 0.197 ± 0.169 | 1.172 | 0.241 |
|  |  |  |  |  |  |
| Native flower cover | beta | **Julian day** | **-0.769 ± 0.010** | **-7.720** | **<0.001** |
|  |  | % sealed area | -0.062 ± 0.084 | -0.733 | 0.464 |
|  |  | **Garden size** | **-0.446 ± 0.094** | **-4.772** | **<0.001** |
|  |  | Green space size | 0.096 ± 0.074 | 1.290 | 0.195 |
|  |  |  |  |  |  |
| Proportion exotic flower cover | beta | **Julian day** | **0.738 ± 0.085** | **8.673** | **<0.001** |
|  |  | % sealed area | -0.221 ± 0.175 | -1.260 | 0.208 |
|  |  | **Garden size** | **-0.379 ± 0.187** | **-2.032** | **0.042** |
|  |  | Green space size | 0.320 ± 0.170 | 1.884 | 0.060 |
|  |  |  |  |  |  |
| *Flower visits* |  |  |  |  |  |
| Total flower visits | Poisson^a^ | **Julian day** | **-1.005 ± 0.102** | **-9.821** | **<0.001** |
|  |  | % sealed area | -0.050 ± 0.148 | -0.337 | 0.736 |
|  |  | Garden size | 0.225 ± 0.159 | 1.412 | 0.158 |
|  |  | Green space size | 0.036 ± 0.142 | 0.252 | 0.801 |
|  |  | Proportion exotic flower cover | 0.113 ± 0.113 | 0.994 | 0.320 |
|  |  |  |  |  |  |
| Visits to exotic flowers | Poisson^a^ | Julian day | -0.030 ± 0.118 | -0.251 | 0.802 |
|  |  | % sealed area | -0.078 ± 0.178 | -0.438 | 0.661 |
|  |  | Garden size | 0.054 ± 0.190 | 0.283 | 0.778 |
|  |  | Green space size | 0.166 ± 0.176 | 0.945 | 0.345 |
|  |  | **Proportion exotic flower cover** | **0.844 ± 0.162** | **5.220** | **<0.001** |
|  |  |  |  |  |  |
| Visits to native flowers | Poisson^a^ | **Julian day** | **-1.055 ± 0.109** | **-9.705** | **<0.001** |
|  |  | % sealed area | -0.093 ± 0.136 | -0.687 | 0.492 |
|  |  | Garden size | 0.230 ± 0.148 | 1.554 | 0.120 |
|  |  | Green space size | 0.023 ± 0.131 | 0.178 | 0.858 |
|  |  | **Proportion exotic flower cover** | **-0.327 ± 0.114** | **-2.868** | **0.004** |
|  |  |  |  |  |  |
| Proportion visits to exotic flowers | binomial^a^ | **Julian day** | **0.653 ± 0.167** | **3.919** | **<0.001** |
|  |  | % sealed area | -0.019 ± 0.196 | 0.098 | 0.922 |
|  |  | Garden size | -0.201 ± 0.214 | -0.942 | 0.346 |
|  |  | Green space size | 0.169 ± 0.197 | 0.859 | 0.390 |
|  |  | **Proportion exotic flower cover** | **1.220 ± 0.178** | **6.840** | **<0.001** |
|  |  |  |  |  |  |
| *Flowering-plant species richness* |  |  |  |  |  |
| Total flowering plant species | Poisson | **Julian day** | **-0.062 ± 0.027** | **-2.019** | **0.032** |
|  |  | % sealed area | 0.024 ± 0.070 | 0.343 | 0.732 |
|  |  | Garden size | 0.006 ± 0.074 | 0.081 | 0.936 |
|  |  | Green space size | 0.093 ± 0.066 | 1.404 | 0.160 |
|  |  | **Total flower cover** | **0.095 ± 0.037** | **2.549** | **0.011** |
|  |  |  |  |  |  |
| Exotic flowering plant species | Poisson | **Julian day** | **0.284 ± 0.053** | **5.342** | **<0.001** |
|  |  | % sealed area | -0.028 ± 0.101 | -0.273 | 0.785 |
|  |  | **Garden size** | **-0.211 ± 0.106** | **-1.986** | **0.047** |
|  |  | **Green space size** | **0.252 ± 0.097** | **2.586** | **0.010** |
|  |  | **Total flower cover** | **0.489 ± 0.051** | **9.629** | **<0.001** |
|  |  |  |  |  |  |
| Native flowering plant species | Poisson | **Julian day** | **-0.196 ± 0.037** | **-5.245** | **<0.001** |
|  |  | % sealed area | 0.041 ± 0.058 | 0.700 | 0.484 |
|  |  | Garden size | 0.095 ± 0.063 | 1.511 | 0.131 |
|  |  | Green space size | -0.045 ± 0.057 | 0.795 | 0.427 |
|  |  | Total flower cover | 0.046 ± 0.041 | 1.128 | 0.259 |
|  |  |  |  |  |  |
| Prop. exotic flow. plant species | binomial | **Julian day** | **0.429 ± 0.055** | **7.775** | **<0.001** |
|  |  | % sealed area | -0.102 ± 0.108 | -0.950 | 0.342 |
|  |  | **Garden size** | **-0.331 ± 0.115** | **-2.866** | **0.004** |
|  |  | **Green space size** | **0.227 ± 0.104** | **2.177** | **0.030** |
|  |  |  |  |  |  |
| *Visited plant species richness* |  |  |  |  |  |
| Total visited plant species | Poisson | **Julian day** | **-0.231 ± 0.046** | **-4.991** | **<0.001** |
|  |  | % sealed area | -0.044 ± 0.098 | -0.452 | 0.651 |
|  |  | Garden size | 0.080 ± 0.105 | 0.767 | 0.443 |
|  |  | Green space size | 0.048 ± 0.094 | 0.508 | 0.612 |
|  |  | **Total flower cover** | **0.121 ± 0.053** | **2.300** | **0.021** |
|  |  |  |  |  |  |
| Exotic visited plant species | Poisson | **Julian day** | **0.271 ± 0.077** | **3.510** | **<0.001** |
|  |  | % sealed area | -0.138 ± 0.101 | -1.366 | 0.172 |
|  |  | Garden size | -0.193 ± 0.106 | -1.825 | 0.068 |
|  |  | Green space size | 0.195 ± 0.101 | 1.937 | 0.053 |
|  |  | **Total flower cover** | **0.411 ± 0.096** | **4.282** | **<0.001** |
|  |  |  |  |  |  |
| Native visited plant species | Poisson | **Julian day** | **-0.471 ± 0.060** | **-7.829** | **<0.001** |
|  |  | % sealed area | -0.021 ± 0.088 | -0.241 | 0.810 |
|  |  | Garden size | 0.164 ± 0.095 | 1.726 | 0.084 |
|  |  | Green space size | 0.004 ± 0.058 | 0.045 | 0.964 |
|  |  | Total flower cover | 0.031 ± 0.058 | 0.525 | 0.599 |
|  |  |  |  |  |  |
| Prop. visited exotic plant species | binomial | **Julian day** | **0.720 ± 0.094** | **7.663** | **<0.001** |
|  |  | % sealed area | -0.148 ± 0.125 | -1.186 | 0.236 |
|  |  | **Garden size** | **-0.415 ± 0.135** | **-3.073** | **0.002** |
|  |  | Green space size | 0.238 ± 0.125 | 1.898 | 0.058 |
|  |  |  |  |  |  |
| Flower-visitor species richness | Poisson^a^ | **Julian day** | **-0.500 ± 0.083** | **-6.041** | **<0.001** |
|  |  | % sealed area | -0.040 ± 0.065 | -0.619 | 0.536 |
|  |  | **Garden size** | **0.153 ± 0.072** | **2.136** | **0.033** |
|  |  | Green space size | 0.011 ± 0.066 | 0.162 | 0.872 |
|  |  | Proportion exotic flower cover | 0.161 ± 0.094 | 1.763 | 0.084 |
|  |  | **Total flower visits** | **0.223 ± 0.059** | **3.792** | **<0.001** |
|  |  |  |  |  |  |
| Proportion non-honey bee visits | binomial^a^ | **Julian day** | **0.664 ± 0.144** | **4.613** | **<0.001** |
|  |  | % sealed area | -0.159 ± 0.126 | -1.261 | 0.207 |
|  |  | Garden size | 0.134 ± 0.137 | 0.975 | 0.329 |
|  |  | Green space size | -0.072 ± 0.124 | -0.585 | 0.558 |
|  |  | Proportion exotic flower cover | -0.131 ± 0.136 | -0.966 | 0.334 |

^a^model with observation-level random effect

**Table S3** Full results for the path model (Fisher’s C=4.94, p=0.293) (see Fig. 4). Estimates give standardized path coefficients ± SE. Significant paths are in bold

| Response | Predictor | Estimate ± SE | | Critical value | p-value |
| --- | --- | --- | --- | --- | --- |
| *Causal path* |  |  | |  |  |
| **Flower-visitor species richness** | **~ Julian day** | **-0.553 ± 0.091** | | **-6.140** | **<0.001** |
| **Flower-visitor species richness** | **~ Proportion flow. exotic species** | **0.276 ± 0.094** | | **2.932** | **0.003** |
| Flower-visitor species richness | ~ Proportion exotic flower cover | -0.098 ± 0.088 | | -1.119 | 0.263 |
| Flower-visitor species richness | ~ Total flow.-plant species richness | 0.067 ± 0.066 | | 1.0167 | 0.309 |
| **Flower-visitor species richness** | **~ Total flower cover** | **0.170 ± 0.074** | | **2.302** | **0.021** |
| **Flower-visitor species richness** | **~ Garden size** | **0.240 ± 0.058** | | **4.115** | **<0.001** |
| **Proportion exotic flower cover** | **~ Julian day** | **0.503 ± 0.063** | | **7.994** | **<0.001** |
| Proportion exotic flower cover | ~ Garden size | -0.073 ± 0.127 | | -0.579 | 0.570 |
| **Proportion flow. exotic species** | **~ Julian day** | **0.476 ± 0.063** | | **7.545** | **<0.001** |
| Proportion flow. exotic species | ~ Garden size | -0.212 ± 0.140 | | -1.517 | 0.146 |
| **Total flow.-plant species richness** | **~ Julian day** | **-0.239 ± 0.071** | | **-3.359** | **0.001** |
| **Total flow.-plant species richness** | **~ Proportion flow. exotic species** | **0.180 ± 0.093** | | **1.948** | **0.054** |
| Total flow.-plant species richness | ~ Proportion exotic flower cover | -0.107 ± 0.094 | | -1.14 | 0.258 |
| **Total flower cover** | **~ Julian day** | **-0.605 ± 0.078** | | **-7.738** | **<0.001** |
| **Total flower cover** | **~ Proportion flow. exotic species** | **0.219 ± 0.101** | | **2.166** | **0.032** |
| Total flower cover | ~ Proportion exotic flower cover | -0.010 ± 0.104 | | -0.017 | 0.986 |
|  |  |  | |  |  |
| *Correlated error* |  |  | |  |  |
| Proportion flow. exotic species | ~~ Proportion exotic flower cover | 0.499 | | 6.482 | <0.001 |
| Total flow.-plant species richness | ~~ Total flower cover | 0.201 | | 2.310 | 0.011 |
|  |  |  | |  |  |
| *D-separation test* |  |  | |  |  |
| Total flow.-plant species richness ~ Julian day + Proportion flow. exotic species + Proportion exotic flower cover + Total flower cover + Garden size | | |  | 0.657 | 0.520 |
| Total flower cover ~ Julian day + Proportion flow. exotic species + Proportion exotic flower cover + Total flow.-plant species richness + Garden size | | |  | 1.468 | 0.163 |

**Table S4** Full model results for lmms testing for the relationships between network indices and the fixed effects (all standardized to mean = 0 and SD = 1; garden size, continuous green space size and total flower visits were log-transformed). P-values are based on Satterthwaite-approximated degrees of freedom (given as subscripts to t-values). Significant p-values are in bold

| Response | Fixed effect | Estimate ± SE | t | p-values |
| --- | --- | --- | --- | --- |
| Shannon interaction diversity | **Julian day** | **2.619 ± 0.383** | **6.838_(102.4)_** | **<0.001** |
|  | **Julian day^2** | **-2.657 ± 0.367** | **-7.250_(101.7)_** | **<0.001** |
|  | Proportion exotic species | -0.010 ± 0.071 | -0.135_(108.9)_ | 0.893 |
|  | % sealed area | -0.070 ± 0.079 | -0.885_(12.9)_ | 0.392 |
|  | Garden size | -0.049 ± 0.091 | -0.539_(17.1)_ | 0.597 |
|  | Green space size | 0.043 ± 0.078 | 0.558_(14.4)_ | 0.586 |
|  | **Total flower visits** | **0.607 ± 0.078** | **7.773_(113.0)_** | **<0.001** |
|  |  |  |  |  |
| Linkage density | **Julian day** | **1.936 ± 0.469** | **4.126_(113.0)_** | **<0.001** |
|  | **Julian day^2** | **-1.841 ± 0.450** | **-4.088_(113.0)_** | **<0.001** |
|  | Proportion exotic species | 0.085 ± 0.080 | 1.060_(113.0)_ | 0.291 |
|  | % sealed area | 0.020 ± 0.067 | 0.302_(113.0)_ | 0.763 |
|  | Garden size | -0.059 ± 0.082 | -0.721_(113.0)_ | 0.472 |
|  | Green space size | 0.051 ± 0.067 | 0.754_(113.0)_ | 0.453 |
|  | **Total flower visits** | 0.727 ± 0.091 | **8.022_(113.0)_** | **<0.001** |
|  |  |  |  |  |
| H2’ | Julian day | 0.025 ± 0.049 | 0.503_(92.0)_ | 0.616 |
|  | Proportion exotic species | -0.031 ± 0.040 | -0.762_(92.0)_ | 0.448 |
|  | % sealed area | -0.033 ± 0.033 | -0.994_(92.0)_ | 0.323 |
|  | Garden size | 0.067 ± 0.043 | 1.578_(92.0)_ | 0.118 |
|  | Green space size | -0.046 ± 0.034 | -1.350_(92.0)_ | 0.180 |
|  | Total flower visits | -0.067 ± 0.049 | -1.350_(92.0)_ | 0.180 |

**
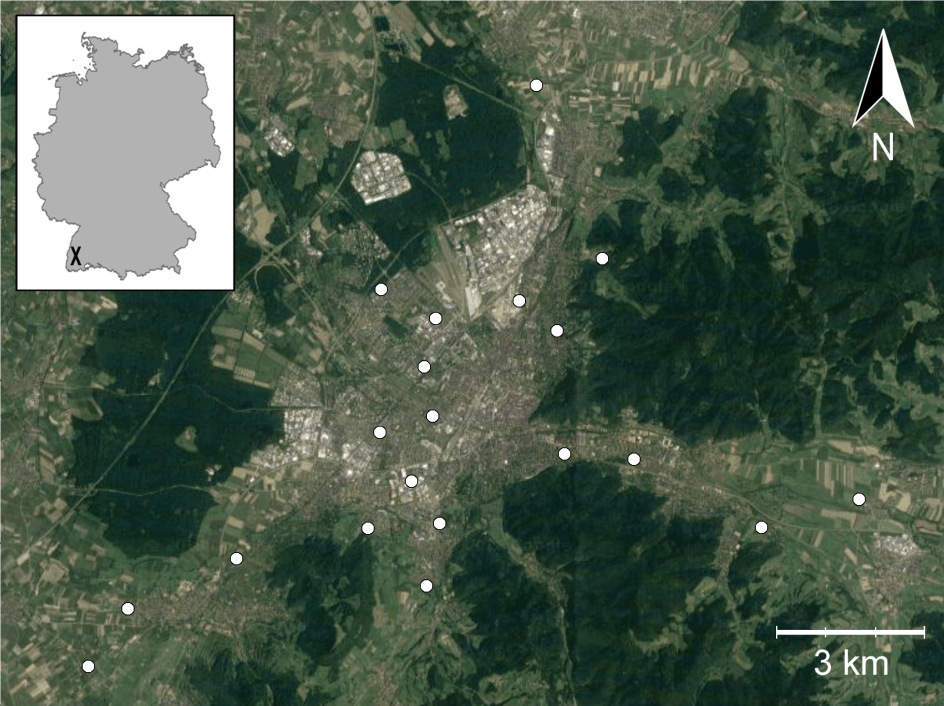
**

**Fig. S1** Map of the study area (centered on the city center of Freiburg) showing the location of all studied gardens in and around Freiburg


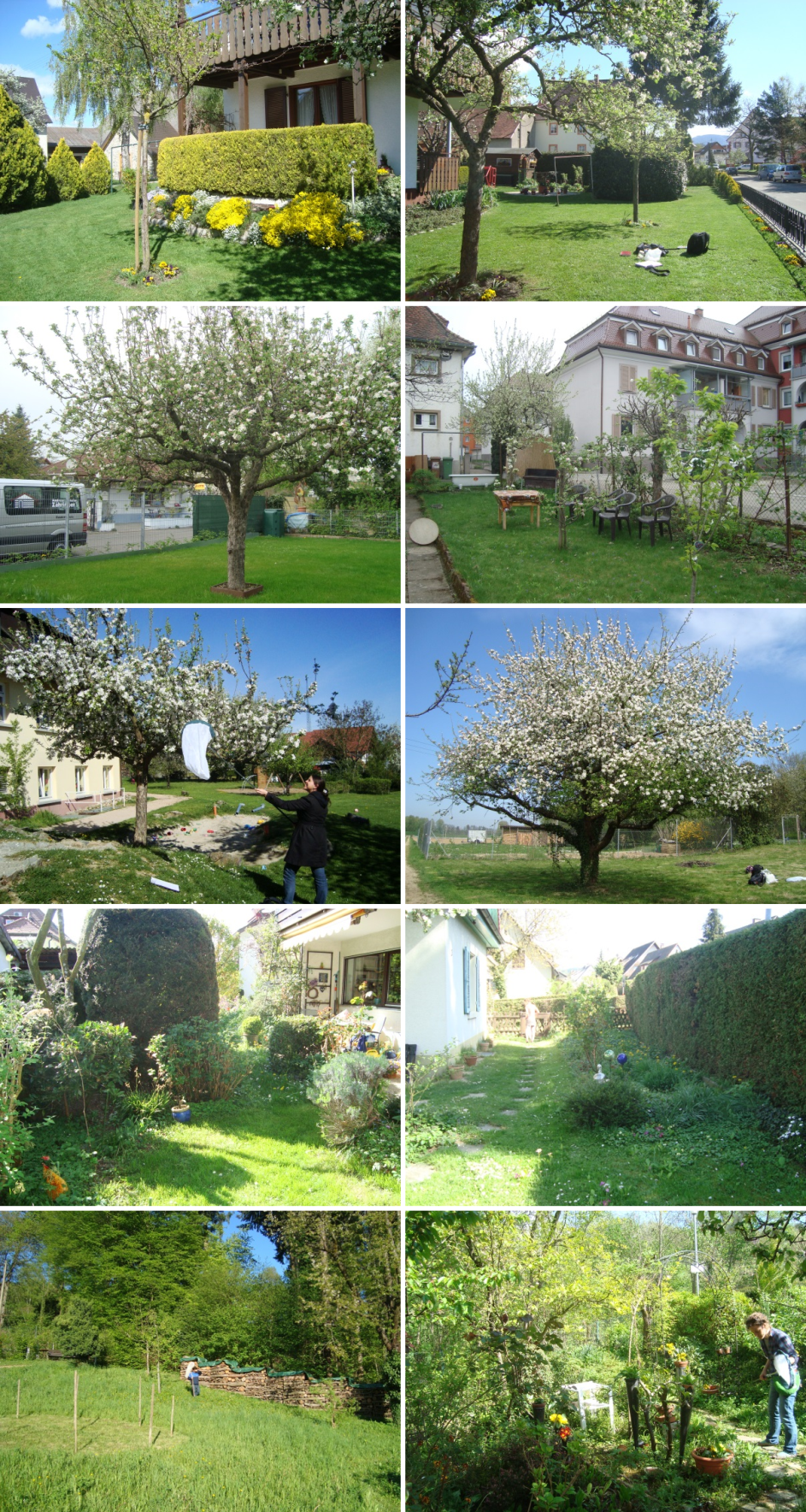


**Fig. S2** Exemplary photographs of the studied gardens. Management and thus flower availability varied among individual gardens


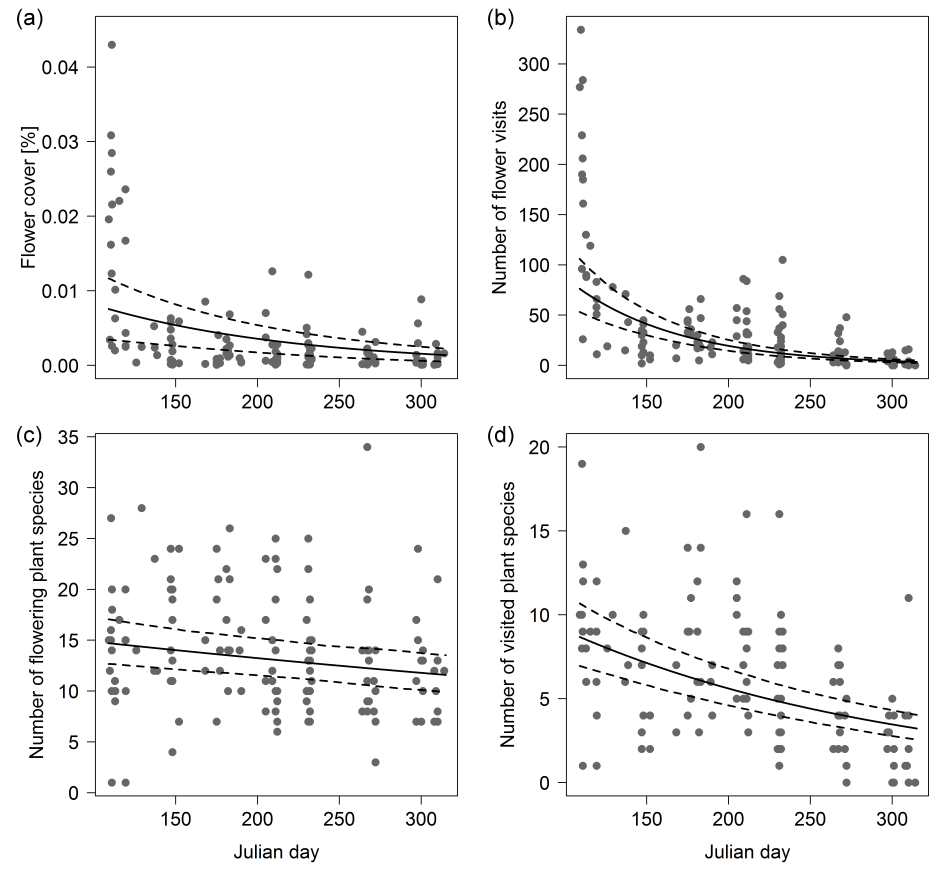


**Fig. S3** Seasonal changes in the total plant communities (native and exotic plants taken together). Total (a) flower cover (proportion of garden area covered by flowers) and (b) total flower visits were highest in spring and decreased over the season. Likewise, (c) the number of flowering plant species (i.e. plants in flower) per garden and especially (d) the number of plant species visited by pollinators decreased. Regression lines indicate the bootstrapped (n=1000) predictions of (a) beta and (b-d) Poisson glmms (p<0.001, except for (c) p<0.01) with 95% CI (dashed lines)


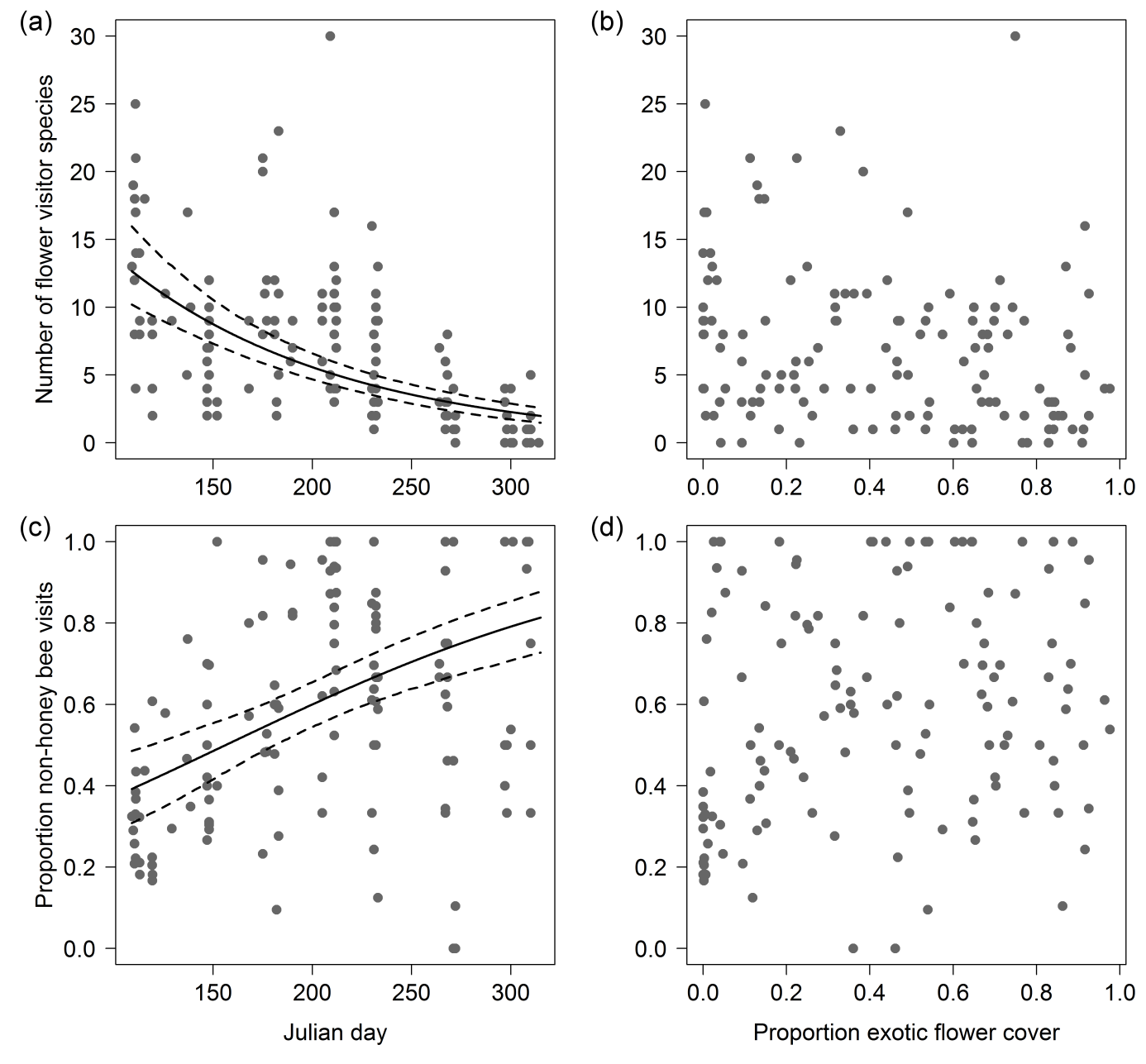


**Fig. S4** Seasonal changes in the total flower-visitor communities. The number of (a) flower visitor species was highest in spring and decreased over the season, but was (b) not statistically related to the proportion of exotic among total flower cover. At the same time, (c) the proportion of flower visits by non-honey bees increased, also (d) without being related to the proportion of exotic flower cover. Regression lines indicate the bootstrapped (n=1000) predictions of significant relationships in glmms (Poisson for flower visitors, binomial for proportion of non-honey bee visits, each p<0.001) with 95% CI (dashed lines)


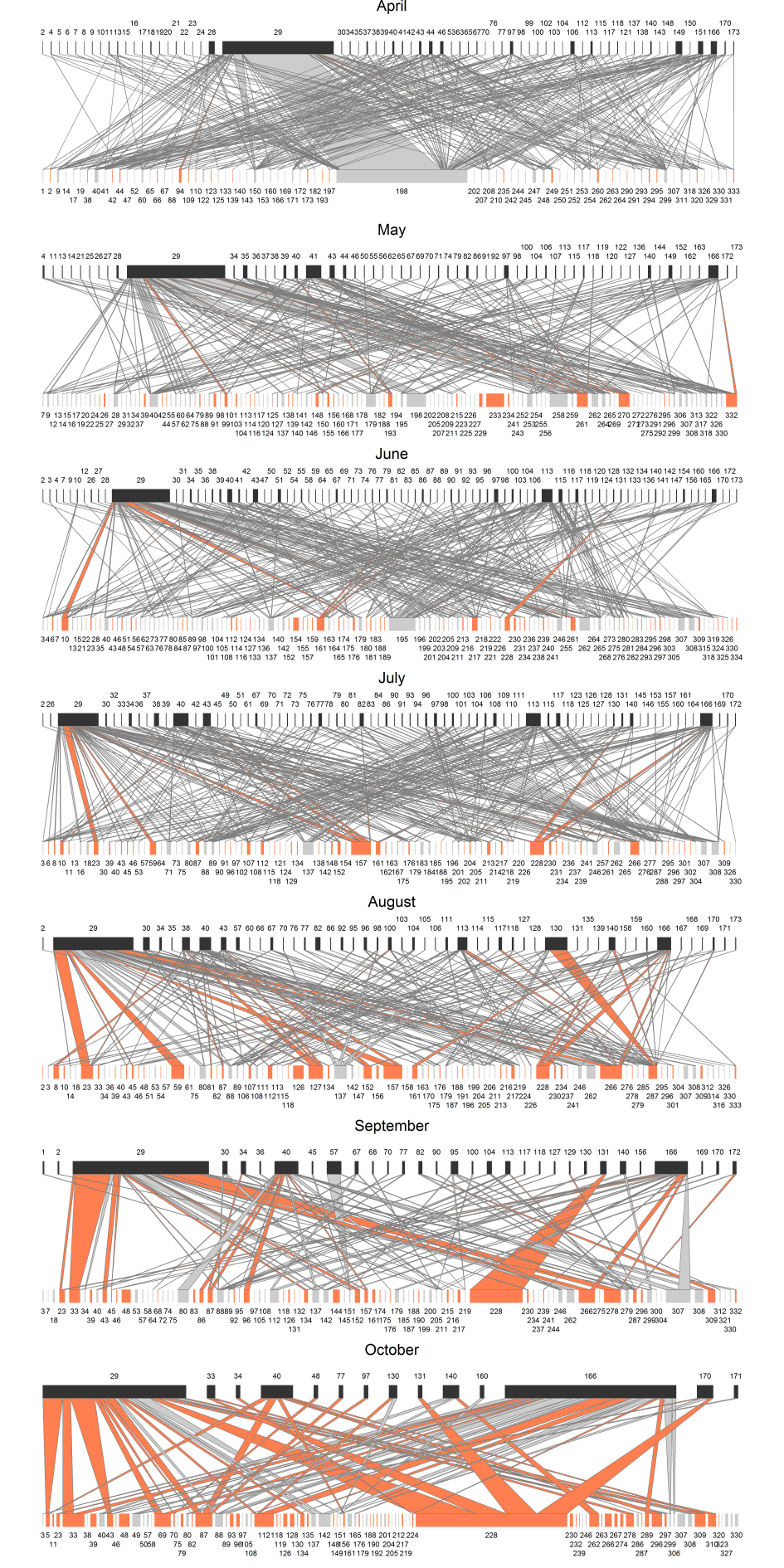


**Fig. S5** Seasonal changes in plant-pollinator networks, based on data pooled for all gardens per month. Width of bars corresponds to flower cover per plant species in the lower and to the number of visits by each pollinator species in the higher level (species identities coded by numbers, see Table S1); width of arrows corresponds to the number of interactions between two species, with the most narrow bars and arrows indication single interactions each (note that the number of interactions varied: April = 2703, May = 525, June = 550, July = 657, August = 532, September = 237, October = 106). Arrows that narrow from top to bottom indicate that a plant species was more often visited than expected solely from the cover of this plant among all plants. In turn, arrows that widen from top to bottom indicate relatively less visited plant species. For plants, light grey bars and arrows indicate interactions of native plant species and red of exotic species, respectively). Non-visited plant species are included but do not have any interactions

**List of the identification keys and further literature used for species identification:**

[Amiet](https://www.researchgate.net/scientific-contributions/2136918060_Felix_Amiet?_sg=30Nhjwl5fvDohA5eW4d3LX0v7NkoXMq08rXw6CKQwpMae-QpNKkqAb1gDbaOOsq1T2t0FDk.r0gDxC80h_nHNfTDvY9B1L-8utRWbQZfQHf7sWVxB26S63t2qdG3wmUe6hyvrBhfyLYz-RlD8dVEfLcz_0dMBw) F, Herrmann M, [Müller](https://www.researchgate.net/profile/Andreas_Mueller28?_sg=30Nhjwl5fvDohA5eW4d3LX0v7NkoXMq08rXw6CKQwpMae-QpNKkqAb1gDbaOOsq1T2t0FDk.r0gDxC80h_nHNfTDvY9B1L-8utRWbQZfQHf7sWVxB26S63t2qdG3wmUe6hyvrBhfyLYz-RlD8dVEfLcz_0dMBw) A, [Neumeyer](https://www.researchgate.net/profile/Rainer_Neumeyer?_sg=30Nhjwl5fvDohA5eW4d3LX0v7NkoXMq08rXw6CKQwpMae-QpNKkqAb1gDbaOOsq1T2t0FDk.r0gDxC80h_nHNfTDvY9B1L-8utRWbQZfQHf7sWVxB26S63t2qdG3wmUe6hyvrBhfyLYz-RlD8dVEfLcz_0dMBw) R (2001) Apidae 3: *Halictus*, *Lasioglossum*. Fauna Helvetica 6. Schweizerisches Zentrum für die Kartografie der Fauna und Schweizerische Entomologische Gesellschaft, Neuchâtel

Amiet F, Herrmann M, Müller A, Neumeyer, R (2004) Apidae 4: *Anthidium*, *Chelostoma*, *Coelioxys*, *Dioxys*, *Heriades*, *Lithurgus*, *Megachile*, *Osmia*, *Stelis*. Fauna Helvetica 9. Schweizerisches Zentrum für die Kartografie der Fauna und Schweizerische Entomologische Gesellschaft, Neuchâtel

Amiet F, Herrmann M, Müller A, Neumeyer R (2007) Apidae 5: *Ammobates*, *Ammobatoides*, *Anthophora*, *Biastes*, *Ceratina*, *Dasypoda*, *Epeoloides*, *Epeolus*, *Eucera*, *Macropis*, *Melecta*, *Melitta*, *Nomada*, *Pasites*, *Tetralonia*, *Thyreus*, *Xylocopa*. Fauna Helvetica 20. Schweizerisches Zentrum für die Kartografie der Fauna und Schweizerische Entomologische Gesellschaft, Neuchâtel

Amiet F, Hermann M, Müller A, Neumeyer R (2010) Apidae 6: *Andrena*, *Melitturga*, *Panurginus*. Fauna Helvetica 26. Schweizerisches Zentrum für die Kartografie der Fauna und Schweizerische Entomologische Gesellschaft, Neuchâtel

Ascher JS, Pickering J (2014) Discover life bee species guide and world checklist (Hymenoptera: Apoidea: Anthophila). http://www.discoverlife.org/mp/20q?guide=Apoidea_species. accessed on 10-Jan-2017

FloraWeb (2018) Daten und Informationen zu Wildpflanzen und zur Vegetation Deutschlands. <http://www.floraweb.de/>. accessed on 10-Aug-2018

Jacobs HJ (2007) Die Grabwespen Deutschlands: Ampulicidae, Sphecidae, Crabonidae: Bestimmungsschlüssel. Goecke & Evers, Keltern

Jäger E J (2011) Rothmaler - Exkursionsflora von Deutschland. Gefäßpflanzen: Grundband, 20th edn. Spektrum, Heidelberg

Kunz PX (1994) Die Goldwespen (Chrysididae) Baden-Württembergs. Landesanstalt für Umweltschutz Baden-Württemberg, Karlsruhe

Margot S, [Golte-Bechtle](https://www.amazon.de/Marianne-Golte-Bechtle/e/B00MBK8OP0/ref=dp_byline_cont_book_2) M, Spohn R (2015) Was blüht denn da? 2nd edn. Franckh Kosmos, Stuttgart
